# Mutational landscape of a chemically-induced mouse model of liver cancer

**DOI:** 10.1016/j.jhep.2018.06.009

**Published:** 2018-10

**Authors:** Frances Connor, Tim F. Rayner, Sarah J. Aitken, Christine Feig, Margus Lukk, Javier Santoyo-Lopez, Duncan T. Odom

**Affiliations:** 1 Cancer Research UK Cambridge Institute, University of Cambridge, Robinson Way, Cambridge CB2 0RE, UK; 2 Department of Histopathology, Addenbrooke’s Hospital, Cambridge University Hospitals NHS Foundation Trust, Hills Road, Cambridge CB2 0QQ, UK; 3 Edinburgh Genomics (Clinical), The Roslin Institute, The University of Edinburgh, Easter Bush, Midlothian EH25 9RG, UK

**Keywords:** Hepatocellular carcinoma, Carcinogen mouse model, Cancer genomics, Mutational signatures, *Hras*

## Abstract

**Background & Aims:**

Carcinogen-induced mouse models of liver cancer are used extensively to study the pathogenesis of the disease and are critical for validating candidate therapeutics. These models can recapitulate molecular and histological features of human disease. However, it is not known if the genomic alterations driving these mouse tumour genomes are comparable to those found in human tumours. Herein, we provide a detailed genomic characterisation of tumours from a commonly used mouse model of hepatocellular carcinoma (HCC).

**Methods:**

We analysed whole exome sequences of liver tumours arising in mice exposed to diethylnitrosamine (DEN). Mutational signatures were compared between liver tumours from DEN-treated and untreated mice, and human HCCs.

**Results:**

DEN-initiated tumours had a high, uniform number of somatic single nucleotide variants (SNVs), with few insertions, deletions or copy number alterations, consistent with the known genotoxic action of DEN. Exposure of hepatocytes to DEN left a reproducible mutational imprint in resulting tumour exomes which we could computationally reconstruct using six known COSMIC mutational signatures. The tumours carried a high diversity of low-incidence, non-synonymous point mutations in many oncogenes and tumour suppressors, reflecting the stochastic introduction of SNVs into the hepatocyte genome by the carcinogen. We identified four recurrently mutated genes that were putative oncogenic drivers of HCC in this model. Every neoplasm carried activating hotspot mutations either in codon 61 of *Hras*, in codon 584 of *Braf* or in codon 254 of *Egfr*. Truncating mutations of *Apc* occurred in 21% of neoplasms, which were exclusively carcinomas supporting a role for deregulation of Wnt/β-catenin signalling in cancer progression.

**Conclusions:**

Our study provides detailed insight into the mutational landscape of tumours arising in a commonly used carcinogen model of HCC, facilitating the future use of this model to better understand the human disease.

**Lay summary:**

Mouse models are widely used to study the biology of cancer and to test potential therapies. Herein, we have described the mutational landscape of tumours arising in a carcinogen-induced mouse model of liver cancer. Since cancer is a disease caused by genomic alterations, information about the patterns and types of mutations in the tumours in this mouse model should facilitate its use to study human liver cancer.

## Highlights

• High burden of stochastic point mutations in carcinogen-induced murine tumours.
• Distinct mutational imprint in tumours arising after diethylnitrosamine exposure.
• Mutations in Ras/MAPK pathway genes drive DEN-initiated liver tumorigenesis.
• *Apc* mutations associated with progression of DEN-initiated tumours to carcinoma.

## Graphical abstract

**Figure:**
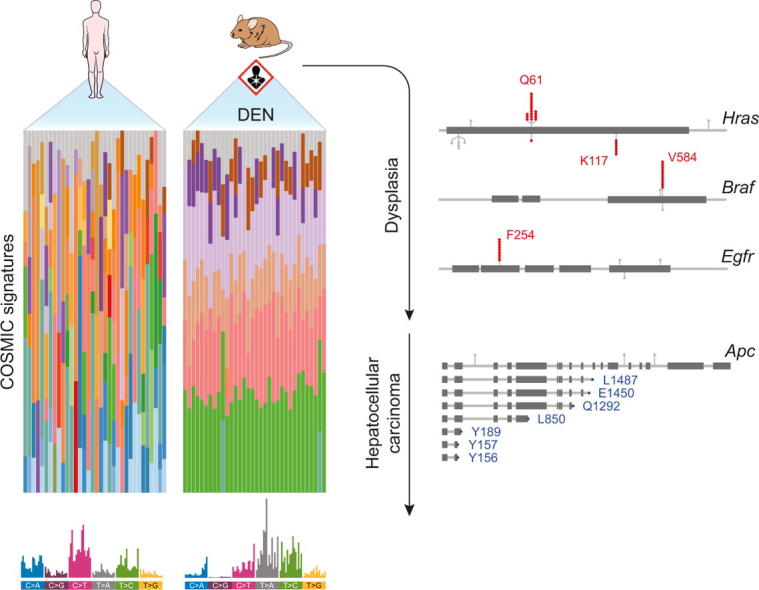

## Introduction

Hepatocellular carcinoma (HCC) is the predominant form of primary liver cancer, which is currently the sixth most frequently diagnosed human cancer. Liver cancer is the second most common cause of cancer death globally and its incidence is increasing in countries with historically low rates.[1], [2] HCC typically develops in the context of end-stage liver disease, resulting from chronic inflammation, fibrosis and cirrhosis, and is almost exclusively caused by environmental risk factors, such as chronic hepatitis virus infection, aflatoxin B exposure, chronic alcohol consumption, and metabolic syndrome.^3^ This diversity of aetiologies appears to be reflected in the molecular heterogeneity of the disease. Over the last few years, next generation sequencing analyses of hundreds of human liver tumours have identified several oncogenic pathways and a wide range of putative driver gene mutations underlying hepatocarcinogenesis.[4], [5], [6], [7], [8], [9]

There are an increasing number of experimental mouse models used in HCC research to study the disease pathogenesis and to assess novel therapeutics.^10^ For several decades, carcinogen-induced tumours have been used in preclinical research, and the most widely used chemical to induce liver cancer in mice is diethylnitrosamine (DEN). When injected into juvenile mice, DEN targets the liver where it is metabolically activated by centrilobular hepatocytes into alkylating agents that can form mutagenic DNA adducts.^11^ The introduction of oncogenic mutations into hepatocytes that are actively proliferating during normal post-natal development can then result in dysplastic lesions which progress to carcinoma. Mouse tumours induced by DEN alone frequently harbour initiating activating mutations in either *Hras* or *Braf* proto-oncogenes.[12], [13] In a related model in which tumours are induced using DEN as an initiator followed by phenobarbital as a tumour promoter, chromosomal instability and activating mutations in β-catenin have been implicated in tumour progression.^14^ There is also evidence that inflammation is a contributing factor to DEN-induced hepatocarcinogenesis. As well as acting as a genotoxin, DEN is also hepatotoxic causing necrotic cell death. This damage triggers an inflammatory response resulting in elevated expression of mitogens, such as interleukin-6, which promote compensatory proliferation of surviving hepatocytes.^15^

No single mouse model can capture all aspects of human HCC, although each can recapitulate at least some of the genetic and/or cellular features of the human disease. For example, a comparison of global gene expression profiles showed that HCC from DEN-treated mice resembles a subclass of human HCC associated with poor prognosis.^16^ However, there are few studies which compare the genome-wide mutational landscapes of mouse cancer models to those seen in the human cancer. Such oncogenomic evaluations will be crucial to identify the most appropriate preclinical mouse model for specific clinical questions. To this end, we describe the exome-wide mutational pattern in tumours arising in the DEN mouse model of HCC, which has, and continues to be, commonly used in preclinical research to understand the biology of liver cancer.

## Materials and methods

### Generation of mouse samples

Male C3H/HeOuJ mice were administered a single intraperitoneal injection of DEN (20 mg/kg body weight) aged 14–16 days. Liver samples were collected during the first 24 h following DEN administration; tumour samples were collected up to 40 weeks after treatment. Untreated C3H male mice were used for reference tissue samples or aged up to 76 weeks for spontaneous liver tumour samples. Tissue samples were snap frozen for DNA extraction and/or fixed in neutral buffered formalin for histological analyses.

### Histological analyses

Histochemical staining with haematoxylin and eosin (H&E) or using the Gomori’s method was carried out on formalin-fixed paraffin-embedded tissue sections. Immunohistochemistry was performed using antibodies against β-catenin (BD Biosciences); phospho-histone H2AX (Merck Millipore); O^6^-ethyl-2-deoxyguanosine (Squarix Biotechnology); and Ki67 (Bethyl Laboratories). Quantification of nuclear staining for O^6^-ethyl-2-deoxyguanosine and phospho-histone H2AX was done using ImageScope software (Leica Biosystems). Tumours were classified according to the International Harmonization of Nomenclature and Diagnostic Criteria for Lesions in Rats and Mice (INHAND) guidelines.^17^

### DNA isolation, whole exome sequencing and sequence alignment

Genomic DNA was isolated from liver tumours and from ear/tail samples using the AllPrep DNA/RNA mini kit or the DNeasy blood & tissue kit (Qiagen), according to the manufacturer’s instructions. Exome capture libraries were prepared following the instructions of the SureSelectXT mouse all exon target enrichment system (Agilent Technologies). Exome libraries were sequenced using a 125 base pair paired-end read protocol on an Illumina HiSeq 2500.

Sequencing reads were aligned to the C3H_HeJ_v1 mouse genome assembly (Ensembl release 90^18^) using BWA (versions 0.6.1 or 0.7.12^19^). Aligned bam files were annotated using Picard tools (version 1.124^20^) and sequencing coverage metrics calculated using samtools (version 1.1^21^). Aligned reads for human samples from the LICA-FR and LIAD-FR cohorts were downloaded from the European Genome-phenome Archive at EMBL-EBI (accessions EGAD00001000131, EGAD00001001096 and EGAD00001000737).

### Variant identification, prioritisation and validation

Single nucleotide and indel variants were called using Strelka (version 1.0.14^22^) and autosomal copy number variants were called using CNVkit (version 0.7.2^23^). Variants were subject to multiple filtering steps, as described in the Supplementary information.

High-likelihood cancer driver genes were prioritised from a list of genes known to harbour *bona fide* cancer driver mutations.^24^ Cancer genes with above expected levels of non-synonymous mutations were identified by fitting the number of observed mutations at each variant locus to a Poisson distribution, and then combining variant loci at the gene level using a multinominal model from the R XNomial package (version 1.0.4^25^). Non-synonymous SNVs in the identified cancer driver genes of interest, *Hras*, *Braf*, *Egfr* and *Apc*, were confirmed using conventional Sanger sequencing or by visual inspection of aligned reads.

### Phylogenetic and mutational signature analyses

A phylogenetic tree was built in R using the ape package (version 3.5^26^). Pairwise distances between samples were calculated as the number of genomic loci at which the sample genotypes differ and trees were constructed using a neighbour-joining algorithm.^27^

For the mutational spectra analysis, SNVs were annotated by the 96 possible trinucleotide context substitutions. The distributions of 5′ and 3′ nucleotides flanking the SNVs were calculated directly from the reference genome. Comparison between human and mouse mutational signatures was facilitated by normalisation of C3H/HeJ nucleotide context distributions using the ratios of known trinucleotide prevalences in C3H/HeJ and human genomes. The proportions of COSMIC mutational signatures^28^ represented in the mutational profile from each sample were calculated using the R package deconstructSigs (version 1.8.0^29^).

For further details regarding the materials and methods used, please refer to the CTAT table and Supplementary information.

## Results

### DEN-initiated carcinogenesis in mouse hepatocytes

**Fig. 1:**
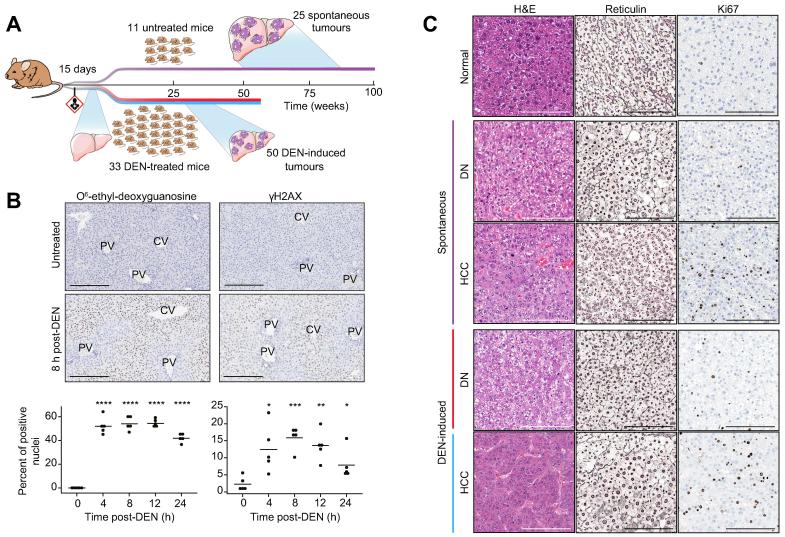
**DEN-initiated carcinogenesis in mouse hepatocytes.** (A) Overview of experimental design. Cohorts of C3H male mice aged 14–16 days were either administered DEN or left untreated. Liver samples were collected during the first 24 h after DEN exposure for histopathological analysis, or mice were aged to develop tumours. Dysplastic nodules and HCCs were collected from cohorts of mice 24–26 weeks and 26–40 weeks, respectively, after administration of DEN; spontaneous tumours were collected from mice aged 37–76 weeks. (B) *In situ* detection and quantification of DEN-induced DNA damage. Representative photomicrographs of immunohistochemistry for O^6^-ethyl-2-deoxyguanosine and gamma-H2AX in untreated mice and 8 h post-DEN injection. All scale bars = 200 μm. Original magnification ×200. Automated image quantification was used to evaluate the dynamics of DNA damage for O^6^-ethyl-2-deoxyguanosine and gamma-H2AX over the course of 24 h (n = 5 samples per timepoint; bar indicates mean; Welch two-sample *t* test: *p <0.05, **p <0.01, ***p <0.001, ****p <0.0001). (C) Histology of murine hepatocellular neoplasms. Representative photomicrographs of serial sections of normal liver tissue and liver tumours arising in DEN-treated and untreated mice. H&E staining demonstrates tissue morphology; reticulin staining is used to assess architecture (normal: staining around each cord of hepatocytes; DN: loss of regular architecture; HCC: thickened trabeculae and corresponding reduction in staining); and Ki67 identifies mitotic cells (normal adult liver is mostly quiescent; DN and HCC show increasing numbers of dividing cells). All scale bars = 200 μm. Original magnification ×200. CV, central vein; DEN, diethylnitrosamine; DN, dysplastic nodule; H&E, haematoxylin and eosin; HCC, hepatocellular carcinoma; PV, portal vein.

We generated chemically-initiated liver tumours using a well-established protocol in which juvenile (14–16 day old) C3H/HeOuJ male mice were administered a single intraperitoneal injection (20 mg/kg body weight) of DEN.^30^ Animals were then aged for up to 40 weeks. The C3H strain was chosen because it is highly susceptible to the development of both treatment-induced and spontaneous liver tumours.^31^ We therefore also aged a cohort of untreated C3H male mice for up to 76 weeks to generate spontaneous liver neoplasms for comparison with the DEN-initiated tumours (Fig. 1A).

DEN primarily targets the liver, in which it is metabolically activated by cytochrome P450 enzymes in hepatocytes.^32^ The resulting DEN metabolites can directly damage DNA by alkylating nucleobases. Of particular interest are the O6 position in guanine and the O4 position in thymine, which are both vulnerable to nucleophilic attack resulting in adducts with the potential to be miscoding.^11^ We examined the immediate DNA damage in 15-day old C3H livers over 24 h after exposure to DEN. Metabolic activation of DEN occurred within 4 h after administration, as seen by the presence of the promutagenic O6-ethyl deoxyguanosine adduct using immunohistochemistry (Fig. 1B). As expected, the majority of positively staining cells were found in centrilobular (zone 3) hepatocytes, consistent with the known high expression of cytochrome P450 enzymes and more extensive drug metabolism by hepatocytes in this region.^33^ As may be expected, DNA double strand breaks also accumulated after DEN treatment, as seen by the rapid accumulation and elimination of phosphorylated histone H2AX over the following 24 h (Fig. 1B).

All DEN-treated mice developed multiple, macroscopically identifiable tumours by 25 weeks after administration, concordant with previous studies.^34^ H&E and reticulin stained tumour tissue sections from DEN-treated and untreated mice (Fig. 1C) were classified by a histopathologist using standardised INHAND diagnostic criteria;^17^ this revealed that all neoplasms had a hepatocellular phenotype. Almost all tumours arising in mice up to 26 weeks after DEN treatment were dysplastic nodules (DNs). Hepatocellular carcinomas were present at later time points, some of which had a nodule-in-nodule appearance, supporting the hypothesis of stepwise progression from DN to HCC.^35^ We did not detect evidence of elevated immune infiltrates in the spontaneous and DEN-induced liver tumours; the leukocyte populations in the DNs and HCCs were low in number, similar to those found in normal liver tissue, as shown by immunohistochemical staining for CD45 (Fig. S1). In addition to the macroscopically dissected tumours, examination of residual liver tissue of DEN-treated mice revealed microscopic basophilic and eosinophilic foci of cellular alteration (data not shown). These localised proliferations of phenotypically distinct hepatocytes represent potential neoplastic precursors to DNs, and in turn HCC.^17^

The development of spontaneous tumours in untreated C3H showed greater histological and temporal variability (37–76 weeks). Importantly, DN and HCC tumours arising in these untreated mice were histologically indistinguishable from those treated with DEN (Fig. 1C). Furthermore, all these murine tumours histopathologically mimic their corresponding human tumours.

### Diversity of somatic SNVs reveals independent evolution of DEN-induced neoplasms

**Fig. 2:**
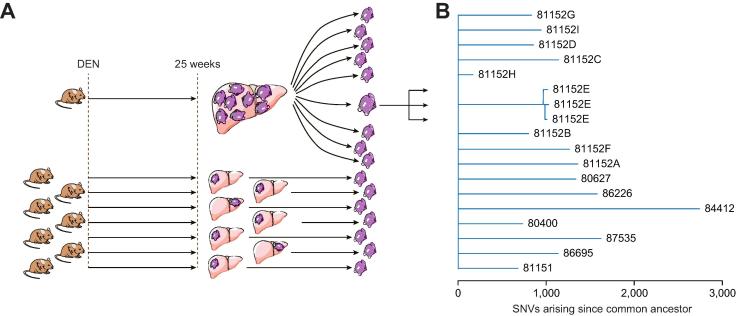
**Independent evolution of DEN-initiated liver tumours is revealed by their unique SNV profiles.** (A) Experimental design. Liver neoplasms were generated by intraperitoneal injection of DEN into 14–16-day old mice, which were then aged for 24–26 weeks. We performed whole exome sequencing of nine separate nodules isolated from a single mouse liver and of single nodules from livers of seven other mice. To evaluate the noise associated with library preparation, triplicate sequencing libraries were prepared in three separate batches for a single nodule. (B) Phylogenetic analysis of DEN-initiated tumours. A phylogenetic tree was constructed using the ape package in R, where branch lengths correspond to the number of unshared SNVs. Long branch lengths indicate no relatedness among the nodules within a single mouse, whereas three replicate libraries from the single tumour had short branches, indicating few SNV differences. Branches are labelled using mouse and tumour identification codes. DEN, diethylnitrosamine; SNV, single nucleotide variant.

Whole exome sequencing was performed on DNA isolated from 50 discrete neoplasms excised from the livers of 33 individual C3H male mice given a single intraperitoneal administration of DEN as juveniles. 34 of the DEN-induced neoplasms were of sufficient size to provide additional tissue for histopathological examination; of these, 16 were classified as DNs and 18 as HCCs. The whole exome sequences of the remaining 16 DEN-induced neoplasms were used only for the phylogenetic analysis (see later and Fig. 2). In addition, whole exome sequencing was performed on DNA isolated from 25 macroscopically visible liver neoplasms (22 DNs and 3 HCCs) found in 11 untreated C3H male mice. The targeted exonic regions were sequenced to an average depth of 380x, with 95% of coding DNA sequences covered at >20-fold. Sequencing data were processed to identify somatic nucleotide substitutions, small insertion and deletion mutations and copy number alterations larger than 10 megabase (Mb).

To test whether multiple tumours within one individual mouse treated with DEN had evolved independently, we constructed a phylogenetic tree to examine how closely related the mutational patterns were among nine nodules isolated from a single liver (Fig. 2). The DNA from one of these nodules was isolated and three separate libraries were generated to perform independent exome sequencing. In addition, seven nodules from seven different animals were also included where DEN-induced mutational patterns must have arisen autonomously. As expected, the three exome SNV profiles generated from the single nodule were almost identical. In contrast, very few of the 24,721 SNVs that we identified across all 16 samples in this cohort were shared between neoplasms. Indeed, the SNV profiles of separate neoplasms isolated from the same liver were as divergent as those isolated from separate mice, suggesting that within this sample set each DEN-induced neoplasm was initiated, and evolved, as an independent tumour.

### Carcinogen-initiated liver tumours have a high SNV burden

**Fig. 3:**
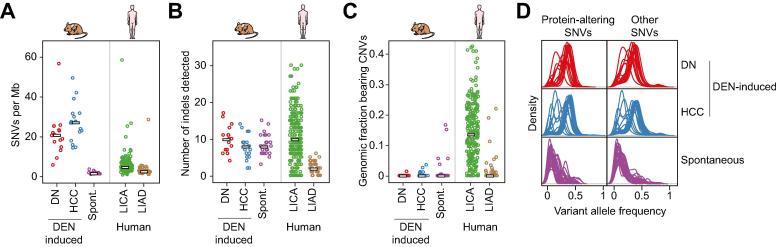
**DEN-initiated neoplasms have a high SNV burden and few indels or copy number variations.** (A) Estimated SNV mutation rates per megabase (Mb) in mouse and human liver tumour cohorts. The point mutation frequencies are shown for DEN-induced DN (n = 16) and HCC (n = 18) and for spontaneous tumours (n = 25) arising in untreated mice. Previously reported human HCC (LICA, n = 224) and hepatic adenoma (LIAD, n = 38) samples are shown for comparison. Each point represents a single sample. Bars indicate the median number of SNVs per Mb. (B) Comparison of frequencies of insertions and deletions (indels, 1–50 base pairs) in each cohort of mouse and human liver tumours. Each cohort had few indels, regardless of tumour histology or aetiology. Bars indicate median number of indels. (C) The fraction of the genome altered by cancer-associated CNVs in each cohort of mouse and human liver tumour samples. Mouse tumours had a lower fraction of their genomes present in CNVs larger than 10 Mb compared with human HCCs (LICA). Bars indicate median genomic fraction with CNVs. (D) Distribution of VAFs in mouse liver tumours. Plots show the density of VAFs in DEN-induced DNs (n = 16) and HCC (n = 18) and in spontaneous tumours arising in untreated mice (n = 25). Each line represents the distribution of VAFs from a single tumour. Separate plots are shown for SNVs classified as either protein-altering or as other (intergenic, intronic, or protein-coding synonymous). DEN-initiated tumours typically have higher VAFs (mean 0.32) than spontaneous neoplasms (mean 0.14). CNV, copy number variant; DEN, diethylnitrosamine; DN, dysplastic nodule; HCC, hepatocellular carcinoma; SNV, single nucleotide variant; VAF, variant allele frequency.

Carcinogen-initiated neoplasms had reproducibly high numbers of somatic SNVs, with an average of 28.4 coverage-independent SNVs per Mb in histologically classified HCCs (Fig. 3A). DNs harboured fewer SNVs on average, albeit still at comparably high numbers (mean 22.1 per Mb). Despite sharing similar histology, the neoplasms which arose spontaneously in untreated mice had much lower SNV burdens, on average 19-fold fewer SNVs per Mb compared with the carcinogen-induced neoplasms. The lower numbers of SNVs in spontaneous tumours is comparable with those seen in human HCC.^6^ By contrast, both murine DEN-induced and spontaneous tumours carried very few somatic indels and copy number variants (Fig. 3B & C). The widespread acquisition of SNVs specifically in the exomes of DEN-induced neoplasms reflects the involvement of a DNA damaging chemical in their pathogenesis.

The different aetiologies of the murine neoplasms may also explain their distinct SNV allele frequencies (Fig. 3D). The SNVs found in DEN-initiated tumours had a much higher variant allele frequency (VAF) than those found in spontaneous tumours (0.32 *vs.* 0.14, on average, *p* value 1.5 × 10^−5^). Spontaneous neoplasms carried many low abundance SNVs, and non-synonymous variants appear to be preferentially selected as a subset of these SNVs had increased VAFs (Fig. 3D). One likely explanation for this is the expansion of cells with acquired driver gene mutations (see later). The uniformly high VAFs in carcinogen-initiated tumours is likely due to the single large burst of mutagenesis upon DEN exposure in the originating cell (Fig. 1B); the consequently high VAF might partially mask later acquisitions of driver mutations, selection and outgrowth of subclones.

### Distinct carcinogen imprint on the exome of DEN-induced neoplasms

**Fig. 4:**
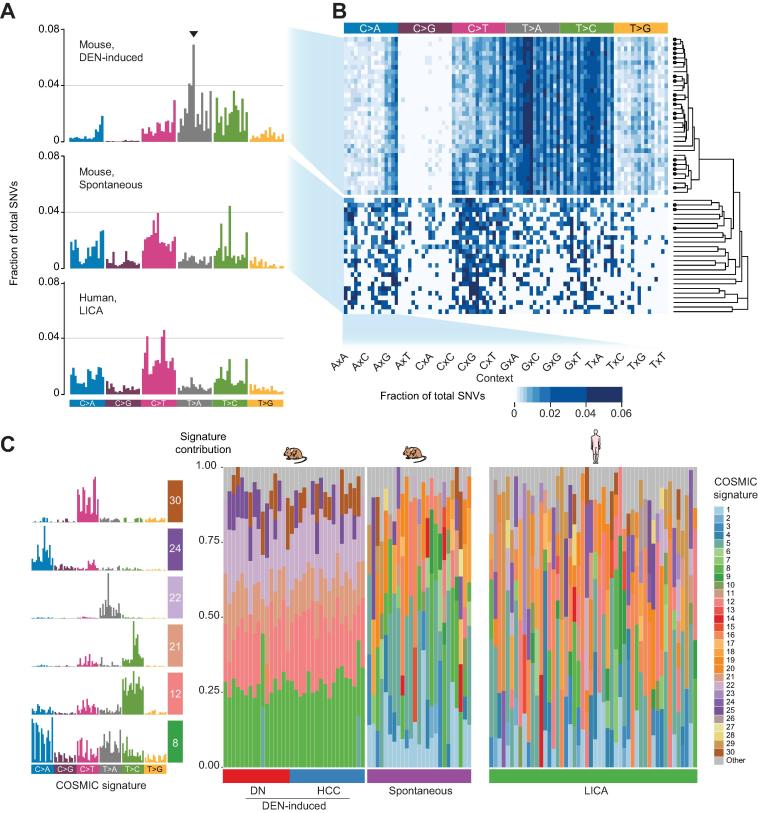
**The exomes of DEN-initiated tumours have distinct and reproducible mutational profiles.** (A) Frequencies of substitution mutations in mouse and human liver tumour cohorts. Mutational profiles are shown for DEN-induced mouse tumours (combined DN and HCC samples, n = 34); for spontaneous tumours (n = 25) arising in untreated mice; and for human liver tumours (LICA, n = 50). The profiles are displayed using the 96-substitution classification, which is defined by reporting the specific base substitution combined with the immediate neighbouring 5′ and 3′ nucleobases. The arrow indicates one example of a trinucleotide context mutational bias observed in DEN-initiated tumours. (B) Heat map of the occurrence of mutational profiles of individual mouse tumours samples (rows) classified by substitution and trinucleotide context (columns). The right panel shows a phylogenetic tree quantifying the clustering observed from the individual mouse sample mutational profiles. A circle indicates an HCC sample; no circle indicates a DN sample. Neoplasms clustered by mutational profile, revealing a clear grouping into DEN-induced *vs.* spontaneous tumours. (C) Mutational portraits of individual mouse and human liver tumours reconstructed using COSMIC mutational signatures. Each column shows the composition of signatures in an individual sample. DEN-induced mouse neoplasms showed reproducible portraits largely composed of six component COSMIC signatures (shown in the left panel). In contrast, mouse spontaneous and human tumour portraits were more heterogeneous. DEN, diethylnitrosamine; DN, dysplastic nodule; HCC, hepatocellular carcinoma.

All categories of somatic base substitutions were found in the exomes of DEN-initiated neoplasms, although C:G to G:C transversions were rarely detected (Fig. 4A; Table 1). Compared with the point mutations seen in untreated mice, DEN exposure resulted in an increase in transition and transversion events at A and T base pairs across the exome. These base substitutions are consistent with the persistence and mutagenicity of unrepaired alkylated thymidine lesions formed by metabolically activated DEN.^11^ T:A to A:T transversions and T:A to C:G transitions have been reported previously as predominant types of mutations induced by DEN, although these studies were limited to the sequencing of specific endogenous cancer genes or of surrogate genes in transgenic mouse mutation assays.[12], [36]

**Table 1:** **Mutation spectra in liver tumours from DEN-treated (n = 34) and untreated (n = 25) mice and in human HCC related to alcohol and metabolic syndrome (LICA) (n = 224).**

**Mutation type**	**Occurrence (%)**		
	**DEN-treated mice**	**Untreated mice**	**Human (LICA)**
Transition			
C:G to T:A	17.2	34.3	42.1
T:A to C:G	29.7	20.9	17.5
Transversion			
C:G to A:T	7.9	21.4	21.1
C:G to G:C	0.8	7.4	6.9
T:A to A:T	37.3	10.7	7.3
T:A to G:C	7.1	5.3	5.1

The 5′ and 3′ nucleobases adjacent to point mutations in mouse DEN-induced tumours showed a complex pattern of biases. For example, we found a distinctive signature in DEN-initiated neoplasms where T:A to A:T transversions occurred more frequently when the T (or A) was preceded by a C (or A) and followed by a T (or G). Hierarchical clustering on the 96 possible trinucleotide substitution contexts showed a consistent mutational pattern shared among both DNs and HCCs arising in C3H inbred mice exposed to DEN (Fig. 4B). We also observed this mutational profile associated with exposure to DEN in liver tumours arising in similarly treated male C57BL/6J mice, a strain reported to be more resistant to liver tumour induction (Fig. S2). Moreover, the mutational patterns of tumours arising spontaneously in untreated C3H male mice clustered separately, highlighting the distinct mutational pattern of the carcinogen-initiated neoplasms (Fig. 4B).

Mathematical modelling of mutational processes in human cancer has defined over 30 mutational signatures, several of which are associated with exposure to specific environmental mutagens.^37^ We used these COSMIC signatures to computationally determine the composition of signatures which most accurately reconstructed the mutational profile of each mouse liver neoplasm.^29^ The resulting mutational portraits of the 34 DEN-initiated neoplasms were notably similar (Fig. 4C). The majority were largely composed of six reported COSMIC signatures: 8, 12, 21, 22, 24 and 30. Interestingly, signatures 12, 22 and 24 have been observed in human liver cancers, with signatures 22 and 24 reported to be associated with exposure to an exogenous mutagen, aristolochic acid or aflatoxin, respectively.[6], [7], [9] The aetiologies of signatures 8, 12, 21 and 30 are currently unknown, although it has been speculated that the transcriptional strand bias reported in signatures 8 and 12 may reflect the involvement of transcription-coupled repair acting on bulky DNA adducts due to exogenous carcinogens.^37^ The six-signature mutational portrait that is characteristic of DEN-induced neoplasms, in both C3H and C57BL/6J strains (Fig. S2), is distinct from the mutational portraits of the 25 tumours arising spontaneously in untreated C3H male mice (Fig. 4C). The latter had a far more heterogeneous composition of individual mutational COSMIC signatures. We also carried out a similar analysis for human HCCs using the exome sequences of 50 randomly selected samples from the ICGC LICA-FR cancer genome project.^6^ The mutational portraits of these human liver cancers also had heterogeneous compositions of individual COSMIC mutational signatures (Fig. 4C). In sum, the mutational portraits of DEN-induced mouse tumours are remarkably homogeneous and reproducible, particularly in comparison with the diversity found within a typical cohort of human HCCs.

### Activating mutation of *Hras* is the most common driver of DEN-induced hepatocarcinogenesis in C3H mice

**Fig. 5:**
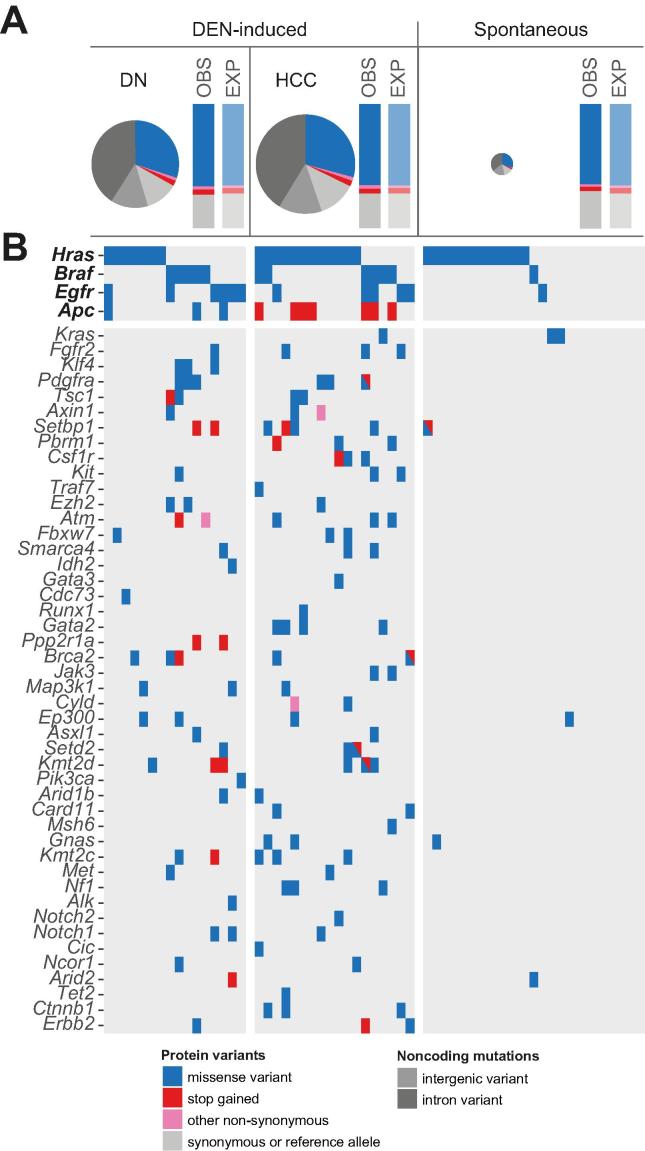
**DEN-initiated and spontaneous mouse liver tumours carry recurrent activating mutations in *Hras*, but only carcinogen-induced tumours acquire a diversity of consequential SNVs in many cancer genes.** (A) Proportions of predicted protein-coding and non-coding variants in DEN-induced and spontaneous liver tumours. Pie charts show the proportions of each variant type in DEN-induced DN (n = 16) and HCC (n = 18) and in spontaneous liver tumours (n = 25). The observed (OBS) distribution of each substitution type was as expected (EXP), regardless of tumour histology or aetiology. The total area of the pie charts reflects the median SNV load within each sample set; spontaneous tumours had extremely low mutational loads. (B) Predicted consequential mutations in oncogenes and tumour suppressor genes for individual tumours. Each column in the table is a mouse tumour sample and each row is a cancer gene showing the occurrence of non-synonymous substitutions found in individual samples. Only genes mutated in at least two samples are shown (see Table S1 for the complete list in each sample of somatic non-synonymous mutations in cancer genes). DEN, diethylnitrosamine; DN, dysplastic nodule; HCC, hepatocellular carcinoma; SNV, single nucleotide variant.

The neoplasms which arose following carcinogen exposure carried a high mutational load in their exomes. For example, each DEN-initiated DN had an average of 583 somatic SNVs in its protein-coding sequence, compared with 26 SNVs in an average spontaneous neoplasm. As expected, 72% of these point mutations are predicted to be non-synonymous, with no detectable bias in the distribution of missense, nonsense and splice site mutations (Fig. 5A). Within our cohort of 34 carcinogen-initiated DNs and HCCs, we have detected potential coding changes in 9,222 genes (data not shown).

**Fig. 6:**
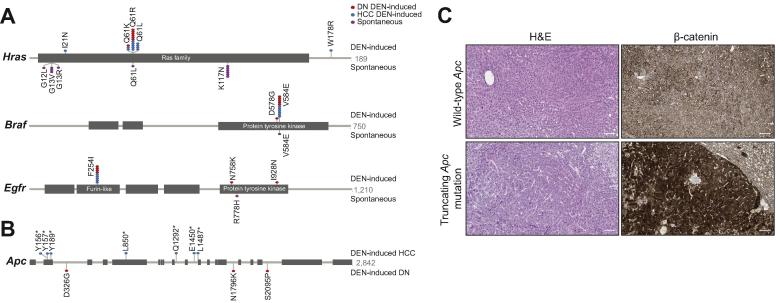
**DEN-initiated and spontaneous mouse liver tumours acquire different recurrent mutations in putative driver genes.** (A) Prevalence and location of somatic mutations in *Hras, Braf* and *Egfr* in DEN-initiated and spontaneous mouse liver tumours. Activating mutations in *Hras* were found at different hotspots in DEN-induced tumours (codon 61) compared with spontaneous tumours arising in untreated C3H mice (codon 117). Hotspot mutations in *Braf* (codon 584) and *Egfr* (codon 254) were recurrent in DEN-induced tumours, in contrast to spontaneous tumours which rarely carried non-synonymous SNVs in *Braf* or *Egfr*. (B) Prevalence and location of somatic mutations in *Apc* in DEN-induced HCCs compared with DEN-induced DNs. Truncating mutations in *Apc* were common in DEN-initiated tumours, exclusively in carcinoma samples. The spontaneous samples did not carry any non-synonymous mutations in *Apc* (not shown, see Table S1). (C) Aberrantly elevated nuclear β-catenin protein expression in tumours with a nonsense mutation in *Apc*. Representative photomicrographs of serial tissue sections of DEN-induced HCCs. H&E staining demonstrates similar tumour morphology in tumours with wild-type *Apc* (upper panels) and in tumours with a nonsense *Apc* mutation (lower panels). Immunohistochemistry for β-catenin protein demonstrates aberrant strongly positive nuclear staining in *Apc* mutant HCC (lower panels). Codon lengths of genes are shown at the right of each gene schematic. All scale bars = 100 μm. Original magnification ×100. DEN, diethylnitrosamine; DN, dysplastic nodule; HCC, hepatocellular carcinoma.

We sought evidence for putative driver genes of hepatocarcinogenesis in our mouse model by searching for enrichment of non-synonymous mutations in validated oncogenes and tumour suppressor genes.^24^ We identified cancer genes which carried non-synonymous mutations more frequently than expected, with additional weight being given to genes which had recurrent hotspot SNVs (Fig. 5B; Table S1). This approach revealed that *Hras* is the predominant, although not obligatory, oncogenic driver of HCC in juvenile male C3H mice that have been administered a single dose of DEN. Over half of the DEN-initiated tumour samples harboured a non-synonymous mutation in the *Hras* proto-oncogene, almost exclusively an activating hotspot mutation in codon 61 (Fig. 6A; Table S2). The most common missense variant in codon 61 caused a glutamine to arginine substitution and was an A:T to G:C transition in the second base, which is consistent with the formation by DEN metabolites of one of the major promutagenic adducts, O4-ethyl-thymine. The incidence of *Hras* mutation increased from 44% in DNs to 67% of HCC samples, suggesting that cells with oncogenic *Hras* had a selective advantage during DEN-initiated hepatocarcinogenesis. The neoplasms which arose in our untreated male C3H mice also had a high prevalence of non-synonymous mutations in *Hras* (48%), although the mutation spectrum was different. Almost half of the point mutations were identical G:C to T:A transversions in codon 117 (Fig. 6A; Table S2), causing a lysine to asparagine substitution which is predicted to activate Ras.^38^

Less frequently occurring oncogenic drivers of DEN-initiated hepatocarcinogenesis in the C3H strain appear to be *Braf* and *Egfr* (Figs. 5B; 6A; Table S2). Almost one-third of tumours carried an identical activating hotspot mutation in *Braf*: an A:T to T:A transversion in codon 584, resulting in a valine to glutamic acid substitution in the kinase domain. We also identified a potentially activating hotspot missense mutation at codon 254 of the extracellular domain of *Egfr* in approximately one-quarter of DEN-initiated tumours. An activating mutation in *Hras*, *Braf* or *Egfr* was present in every DEN-initiated neoplasm, although these mutations were very rarely found together in the same tumour. This apparent mutual exclusivity is likely because they can replace each other in terms of their oncogenic potential. The driver mutation spectra in DEN-induced liver tumours is reported to be influenced by the strain background of the mouse model.^13^ Indeed, we have also observed that the same induction protocol used in the C57BL/6J strain results in tumours which predominantly carry an activating hotspot mutation in *Braf* rather than in *Hras* (Fig. S3).

Every carcinogen-induced tumour carried non-synonymous SNVs in several *bona fide* cancer genes: on average we detected non-synonymous SNVs in five oncogenes and/or tumour suppressor genes in DEN-initiated tumours (range 1–11) (Table S1). This considerable diversity of cancer genes that were mutated at low frequency after exposure to DEN limited our ability to detect any commonly mutated secondary drivers of DEN-initiated hepatocarcinogenesis. Nevertheless, we observed truncating mutations in *Apc* in 39% of HCC samples; nonsense mutations in *Apc* were not detected in the cohort of DNs (Figs. 5B; 6B; Table S2). The cancers bearing *Apc*-truncating mutations all showed aberrantly elevated levels of nuclear β-catenin (Fig. 6C), suggesting that loss of *Apc* function and disruption of the canonical Wnt/β-catenin pathway can play a role in the progression to carcinoma in this model.

By comparison, spontaneous tumours from untreated mice contained few detectable point mutations in cancer genes (Fig. 5B; Table S1). As previously discussed, approximately half were potentially driven by missense mutations activating the Ras signal transduction pathway. However, we could not unequivocally propose a driver gene for the remaining samples. The failure to detect other subtle mutations in potential driver genes may reflect a polyclonal composition of the spontaneous tumours and/or the involvement of other types of genetic or epigenetic alterations during tumorigenesis.

We used a pathway analysis approach to assess how well the DEN mouse model recapitulates human HCC, as defined by cancer-associated gene mutations. Tumour samples were annotated with a list of Reactome pathways that contained a mutated cancer-associated gene(s). Based on these mutated pathways murine liver tumours, both DEN-induced and those arising in untreated C3H mice, clearly clustered separately from human HCCs (Fig. S4).

## Discussion

Chemically-induced mouse models of liver cancer are important tools widely used to study the molecular pathogenesis of human HCC.^34^ Over the last decade, large scale sequencing analyses of patient tumour samples have produced detailed profiles of the genetic aberrations found in human liver cancer genomes.[4], [5], [6], [7], [8], [9] It is important now to have similar descriptions of the genomic landscapes of the experimental mouse models used to inform the human disease.[39], [40] Here we have described the mutational landscape of one of the most frequently used models of HCC, in which liver cancer is induced by a single injection of the genotoxin DEN into juvenile male mice.

Our strategy comparing spontaneously occurring liver tumours with those initiated by exposure to DEN allowed the direct comparison of the histopathology and genomic impact of carcinogen exposure. By controlling the initiating carcinogenic event the liver lesions in DEN-treated mice developed from early DNs to carcinoma within a short, relatively consistent timeframe. The liver lesions arising in untreated mice arose at a much lower incidence and with a longer, more variable latency. Based on their histological appearance, liver tumours resulting from exposure to DEN were indistinguishable from those that arose spontaneously in untreated mice. This result parallels that found in human liver tumours, where heterogeneous molecular phenotypes can underlie HCC samples that are histologically similar. Indeed, these murine dysplastic lesions and carcinomas mimicked the histological features of their corresponding human tumours. In sharp contrast, however, this similarity was not seen in mutational landscapes: the exomes of DEN-induced neoplasms clearly reflected the DNA damage caused by chemical carcinogenesis.

DEN-induced tumours carried a notably high burden of somatic mutations, which allowed us to demonstrate that multiple tumours can evolve independently within an individual liver. We did not identify evidence of metastatic clones within a liver, although this may reflect the small sample size. The mutational frequencies were much higher than those seen in most human solid tumours, including HCC. Perhaps not surprisingly, human lung and skin cancers that result from environmental exposure to potent mutagens are among the few human tumour types with mutational burdens similar to those we report here.^24^ Almost all of the DNA changes in the DEN-induced tumours were single base substitutions, consistent with the genotoxic action of DEN.^11^ Indeed, we confirmed that one of the major pro-mutagenic adducts caused by short-lived DEN metabolites was generated rapidly in centrilobular hepatocytes. It is likely that most of the genetic damage in tumours arising in livers exposed to DEN occurs when the originating hepatocytes are exposed to the carcinogen. In contrast to the elevated SNV levels, we did not find any evidence that DEN-induced cancer genomes have gross widespread alterations in chromosomal structure; we detected very few insertions, deletions or copy number variants in the exomes of DEN-induced tumours. This combination of a high exome-wide SNV burden with a paucity of copy number alterations has been observed in human cancers,^41^ as well as other carcinogen-induced mouse models of cancer.^39^

Exposure to DEN left a common mutational imprint in the tumour exomes of treated mice. Indeed, the same small subset of reported signatures of mutational processes was readily identified computationally in every DEN-induced tumour. Notwithstanding this common imprint, each individual exome, including those of neoplasms arising within the same liver, carried a unique combination of somatic base substitutions. The majority of these SNVs are likely to be passenger mutations. However, we could identify four recurrently mutated genes that are putative oncogenic drivers of HCC in DEN-treated C3H male mice: *Hras*, *Braf*, *Egfr* and *Apc*.

The main genetic trait of DEN-initiated tumours is acquisition of mutations which deregulate signalling cascades involved in cell proliferation and survival. Over 80% of DEN-initiated tumour samples carried an activating hotspot driver mutation in either *Hras* or *Braf*. The remaining ∼20% of samples carried a potentially activating hotspot mutation in *Egfr*, one of the upstream receptor tyrosine kinases that can regulate the Ras signalling pathway. This suggests that constitutive activation of the Ras/Raf/MEK/ERK signal transduction pathway is a hallmark feature in this mouse model of liver cancer.

Activation of the *Hras* proto-oncogene is frequently reported in both spontaneous and chemically-induced liver tumours in mice.^12^ However, the incidence and spectrum of *Hras* mutations is strongly influenced both by the mouse strain used, as well as by the type and dose of chemical and experimental induction protocol employed. Indeed, even between spontaneous and treatment-induced tumours in C3H mice, we observed a difference in the location of the hotspot activating mutation in *Hras* (codon 117 *vs.* codon 61, respectively). The mutational activation of *Braf* is also reported to be influenced by the mouse strain and appears to be related to the strain’s susceptibility to hepatocarcinogenesis.^13^ As expected, we observed a lower frequency of mutations in *Braf* in the DEN-initiated liver tumours in the highly susceptible C3H mouse strain compared to those arising in the more resistant C57BL/6J strain. Changing the strain in the DEN model can therefore be used to increase the probability of a specific driver mutation in the resulting tumour, although the same signalling pathway will likely be affected. This study and others have shown that dysregulation of the Ras/Raf/MEK/ERK pathway is a common route to hepatocarcinogenesis in mice, especially in genotoxic models.[13], [31]

*Apc* is a putative, although not obligatory, gatekeeper of malignant transformation in the DEN liver cancer model; we detected a significant recurrence of *Apc*-truncating mutations exclusively in carcinoma samples and absent in the DNs arising in DEN-treated C3H mice. Activating β-catenin (*Ctnnb1*) mutations have previously been implicated in progression to carcinoma, but in a two-stage model where DEN is given as the initiator followed by treatment with phenobarbital as a tumour promoter.^14^ In contrast with other reports using mice treated with DEN alone (that is, in the absence of a promoter),^42^ we did observe (i) disruption of the canonical Wnt/β-catenin pathway, and (ii) that this disruption was caused primarily by loss-of-function mutations in *Apc* and consequent aberrant nuclear expression of β-catenin.

Aside from these four driver genes, there were no other *bona fide* cancer genes that were recurrently mutated with significance in the set of DEN-initiated tumour samples from this study. Instead, we saw a diversity of low-incidence, non-synonymous point mutations in numerous oncogenes and tumour suppressors, consistent with the known mechanism of mutagenesis by DEN. Specifically, the introduction of a large mutagenic SNV burden stochastically across the genome resulted in heterogeneity at the level of driver gene mutations in the resulting individual tumours. However, it is also possible that common driver genes could have been dysregulated by alternative genetic or epigenetic processes during tumorigenesis.

There are currently several mouse models of liver cancer, each of which recapitulates specific genetic, molecular, and/or histological features of the human disease.^43^ This study highlights several characteristics of the widely used DEN model which can be taken into consideration when selecting an experimental model of HCC, in particular one for use in preclinical research. Tumour initiation in the livers of DEN-treated mice occurs in the context of acute DNA damage; this does not recapitulate the common clinical presentation of human HCC which typically arises from chronic inflammatory liver disease causing fibrosis and cirrhosis.^3^ As a consequence of exposure to the DNA damaging agent DEN there is a widespread introduction of single base variants into the hepatocyte genomes. The resulting liver tumours carry this distinct, reproducible mutational imprint left by DEN and have a burden of mutations that is much higher than that observed in human HCC samples. Furthermore, activating mutations in *Hras*, *Braf* or *Egfr*, which recurrently occur at a high frequency in the DEN-induced liver tumours, are rarely observed in cases of human HCC. The most common cellular processes and pathways implicated in the pathogenesis of human HCC are telomere maintenance, WNT/β-catenin signalling and p53 cell cycle control.[3], [4], [5] However, although mutations in *RAS* family members are rare, a subset of HCC cases have been reported to have aberrantly activated RAS-MAPK signalling which correlates with a poor prognosis.^44^ The DEN-initiated mouse model may replicate the RAS/MAPK signalling dysregulation implicated in this subset of human HCC. One of the common features of human HCC is perturbation of WNT/β-catenin signalling and we also observe disruption of this pathway in the progression to carcinoma in the DEN mouse model. However, the underlying mutations are different between species; activating *CTNNB1* mutations are frequently observed in human HCC samples, while loss-of function mutations in *Apc* were found in mouse DEN carcinomas.

Our study demonstrates how the application of exome sequencing on carefully designed cohorts can reveal novel insights into widely used mouse models of liver cancer. Such oncogenomic descriptions will deepen our understanding of the advantages and limitations of preclinical *in vivo* models and thereby inform the selection of the most appropriate models to study human liver cancer.

## Financial support

This research was supported by Cancer Research UK (core award 20412 and strategic award 22398; F.C., T.F.R., S.J.A., C.F., M.L., D.T.O.), the Wellcome Trust (106563/Z/14/A; S.J.A.) and the European Research Council (615584; F.C., T.F.R., C.F., D.T.O.).

## Conflict of interest

The authors declare no conflicts of interest that pertain to this work.

Please refer to the accompanying ICMJE disclosure forms for further details.

## Authors’ contributions

Study design: FC, TFR, SJA, CF, ML, DTO. Experiments: FC, SJA, CF. Data curation: ML, TFR. Computational analyses: TFR. Interpretation of data: FC, TFR, SJA, CF, DTO. Study concept and writing the draft manuscript: FC. Data visualisation for the manuscript: TFR, SJA, CF. Critical revision of the manuscript: FC, TFR, SJA, DTO. Provision of whole genome sequencing: JSL. Acquisition of funding: DTO.

## References

[1] Torre L.A., Bray F., Siegel R.L., Ferlay J., Lortet-Tieulent J., Jemal A. (2015). Global cancer statistics, 2012. CA Cancer J Clin.

[2] Koh J.C., Loo W.M., Goh K.L., Sugano K., Chan W.K., Chiu W.Y. (2016). Asian consensus on the relationship between obesity and gastrointestinal and liver diseases. J Gastroenterol Hepatol.

[3] Llovet J.M., Zucman-Rossi J., Pikarsky E., Sangro B., Schwartz M., Sherman M. (2016). Hepatocellular carcinoma. Nat Rev Dis Primers.

[4] Schulze K., Nault J.C., Villanueva A. (2016). Genetic profiling of hepatocellular carcinoma using next-generation sequencing. J Hepatol.

[5] Zucman-Rossi J., Villanueva A., Nault J.C., Llovet J.M. (2015). Genetic landscape and biomarkers of hepatocellular carcinoma. Gastroenterology.

[6] Schulze K., Imbeaud S., Letouze E., Alexandrov L.B., Calderaro J., Rebouissou S. (2015). Exome sequencing of hepatocellular carcinomas identifies new mutational signatures and potential therapeutic targets. Nat Genet.

[7] Fujimoto A., Furuta M., Totoki Y., Tsunoda T., Kato M., Shiraishi Y. (2016). Whole-genome mutational landscape and characterization of noncoding and structural mutations in liver cancer. Nat Genet.

[8] Cancer Genome Atlas Research Network (2017). Comprehensive and integrative genomic characterization of hepatocellular carcinoma. Cell.

[9] Letouze E., Shinde J., Renault V., Couchy G., Blanc J.F., Tubacher E. (2017). Mutational signatures reveal the dynamic interplay of risk factors and cellular processes during liver tumorigenesis. Nat Commun.

[10] Bakiri L., Wagner E.F. (2013). Mouse models for liver cancer. Mol Oncol.

[11] Verna L., Whysner J., Williams G.M. (1996). N-nitrosodiethylamine mechanistic data and risk assessment: bioactivation, DNA-adduct formation, mutagenicity, and tumor initiation. Pharmacol Ther.

[12] Maronpot R.R., Fox T., Malarkey D.E., Goldsworthy T.L. (1995). Mutations in the ras proto-oncogene: clues to etiology and molecular pathogenesis of mouse liver tumors. Toxicology.

[13] Buchmann A., Karcier Z., Schmid B., Strathmann J., Schwarz M. (2008). Differential selection for B-raf and Ha-ras mutated liver tumors in mice with high and low susceptibility to hepatocarcinogenesis. Mutat Res.

[14] Aleksic K., Lackner C., Geigl J.B., Schwarz M., Auer M., Ulz P. (2011). Evolution of genomic instability in diethylnitrosamine-induced hepatocarcinogenesis in mice. Hepatology.

[15] Naugler W.E., Sakurai T., Kim S., Maeda S., Kim K., Elsharkawy A.M. (2007). Gender disparity in liver cancer due to sex differences in MyD88-dependent IL-6 production. Science.

[16] Lee J.S., Chu I.S., Mikaelyan A., Calvisi D.F., Heo J., Reddy J.K. (2004). Application of comparative functional genomics to identify best-fit mouse models to study human cancer. Nat Genet.

[17] Thoolen B., Maronpot R.R., Harada T., Nyska A., Rousseaux C., Nolte T. (2010). Proliferative and nonproliferative lesions of the rat and mouse hepatobiliary system. Toxicol Pathol.

[18] Yates A., Akanni W., Amode M.R., Barrell D., Billis K., Carvalho-Silva D. (2016). Ensembl 2016. Nucleic Acids Res.

[19] Li H., Durbin R. (2010). Fast and accurate long-read alignment with Burrows-Wheeler transform. Bioinformatics.

[20] Picard Tools. [cited 2018]. Available from: http://broadinstitute.github.io/picard.

[21] Li H., Handsaker B., Wysoker A., Fennell T., Ruan J., Homer N. (2009). The sequence alignment/map format and SAMtools. Bioinformatics.

[22] Saunders C.T., Wong W.S., Swamy S., Becq J., Murray L.J., Cheetham R.K. (2012). Strelka: accurate somatic small-variant calling from sequenced tumor-normal sample pairs. Bioinformatics.

[23] Talevich E., Shain A.H., Botton T., Bastian B.C. (2016). CNVkit: genome-wide copy number detection and visualization from targeted DNA sequencing. PLoS Comput Biol.

[24] Vogelstein B., Papadopoulos N., Velculescu V.E., Zhou S., Diaz L.A., Kinzler K.W. (2013). Cancer genome landscapes. Science.

[25] XNomial. [cited 2018]. Available from: https://CRAN.R-project.org/package=XNomial.

[26] Paradis E., Claude J., Strimmer K. (2004). APE: analyses of phylogenetics and evolution in R language. Bioinformatics.

[27] Saitou N., Nei M. (1987). The neighbor-joining method: a new method for reconstructing phylogenetic trees. Mol Biol Evol.

[28] Forbes S.A., Beare D., Boutselakis H., Bamford S., Bindal N., Tate J. (2017). COSMIC: somatic cancer genetics at high-resolution. Nucleic Acids Res.

[29] Rosenthal R., McGranahan N., Herrero J., Taylor B.S., Swanton C. (2016). DeconstructSigs: delineating mutational processes in single tumors distinguishes DNA repair deficiencies and patterns of carcinoma evolution. Genome Biol.

[30] Tolba R., Kraus T., Liedtke C., Schwarz M., Weiskirchen R. (2015). Diethylnitrosamine (DEN)-induced carcinogenic liver injury in mice. Lab Anim.

[31] Maronpot R.R. (2009). Biological basis of differential susceptibility to hepatocarcinogenesis among mouse strains. J Toxicol Pathol.

[32] Kang J.S., Wanibuchi H., Morimura K., Gonzalez F.J., Fukushima S. (2007). Role of CYP2E1 in diethylnitrosamine-induced hepatocarcinogenesis in vivo. Cancer Res.

[33] Oinonen T., Lindros K.O. (1998). Zonation of hepatic cytochrome P-450 expression and regulation. Biochem J.

[34] Heindryckx F., Colle I., Van Vlierberghe H. (2009). Experimental mouse models for hepatocellular carcinoma research. Int J Exp Pathol.

[35] Nam S.W., Park J.Y., Ramasamy A., Shevade S., Islam A., Long P.M. (2005). Molecular changes from dysplastic nodule to hepatocellular carcinoma through gene expression profiling. Hepatology.

[36] Nohmi T., Suzuki T., Masumura K. (2000). Recent advances in the protocols of transgenic mouse mutation assays. Mutat Res.

[37] Alexandrov L.B., Nik-Zainal S., Wedge D.C., Aparicio S.A., Behjati S., Biankin A.V. (2013). Signatures of mutational processes in human cancer. Nature.

[38] Baker R., Wilkerson E.M., Sumita K., Isom D.G., Sasaki A.T., Dohlman H.G. (2013). Differences in the regulation of K-Ras and H-Ras isoforms by monoubiquitination. J Biol Chem.

[39] Westcott P.M., Halliwill K.D., To M.D., Rashid M., Rust A.G., Keane T.M. (2015). The mutational landscapes of genetic and chemical models of Kras-driven lung cancer. Nature.

[40] Nassar D., Latil M., Boeckx B., Lambrechts D., Blanpain C. (2015). Genomic landscape of carcinogen-induced and genetically induced mouse skin squamous cell carcinoma. Nat Med.

[41] Ciriello G., Miller M.L., Aksoy B.A., Senbabaoglu Y., Schultz N., Sander C. (2013). Emerging landscape of oncogenic signatures across human cancers. Nat Genet.

[42] Stahl S., Ittrich C., Marx-Stoelting P., Kohle C., Altug-Teber O., Riess O. (2005). Genotype-phenotype relationships in hepatocellular tumors from mice and man. Hepatology.

[43] Caviglia J.M., Schwabe R.F. (2015). Mouse models of liver cancer. Methods Mol Biol.

[44] Delire B., Starkel P. (2015). The Ras/MAPK pathway and hepatocarcinoma: pathogenesis and therapeutic implications. Eur J Clin Invest.

