# Supplementary material for: Mutational landscape of a chemically-induced mouse model of liver cancer
Source: J Hepatol. 2018 Oct;69(4):840–50. doi: 10.1016/j.jhep.2018.06.009 (PMC6142872; doi:10.1016/j.jhep.2018.06.009)
Supplement: Supplementary data 4 [file CTAT_table.pdf]

EASL | JOURNAL OF HEPATOLOGY

Journal of Hepatology

CTAT methods

Tables for a "Complete, Transparent, Accurate and Timely account" (CTAT) are now mandatory for all revised submissions. The aim is to enhance the reproducibility of methods.

- Only include the parts relevant to your study
- Refer to the CTAT in the main text as 'Supplementary CTAT Table'
- Do not add subheadings
- Add as many rows as needed to include all information
- Only include one item per row

If the CTAT form is not relevant to your study, please outline the reasons why:

1.1 Antibodies

Name	Citation	Supplier	Cat no.	Clone no.
Anti-β-Catenin		BD Biosciences	610154	14
Anti-phospho-Histone H2A.X (Ser139)		Merck Millipore	MABE205	EP854(2)Y
Anti-O6-ethyl-2-deoxyguanosine		Squarix Biotechnology	SQM001.1	ER 6
Anti-Ki67		Bethyl Laboratories Inc.	IHC-00375	
Anti-B220 /CD45R		R&D Systems	MAB1217	RA3-6B2

1.2 Cell lines

Name	Citation	Supplier	Cat no.	Passage no.	Authentication test method

1.3 Organisms

Name	Citation	Supplier	Strain	Sex	Age	Overall n number
Mouse		Charles River	C3H/HeOuJ	male	14 days – 76 weeks	70
Mouse		Charles River	C57BL/6J	male	38 weeks	6

1.4 Sequence based reagents

Name	Sequence	Supplier
Hras ForC1	CTGAAGGTGCCACAGGTGAT	Sigma-Aldrich
Hras RevC2	GAATTGGGGGAGCAAGGACA	Sigma-Aldrich
HrasQ61 ForJ	TCTGTGGATTCTCTGGTCTG	Sigma-Aldrich

HrasQ61 RevK	TGTACTGATGGATGTCCTCG	Sigma-Aldrich
HrasQ61 ForB1	CCCCACTAAGCCGTGTTGTTTTGC	Sigma-Aldrich
HrasQ61 RevB2	TCAGTGTGCACACGGAACCTTCCT	Sigma-Aldrich
HrasQ12/G13/I21 ForH	TTGGCTAAGTGTGCTTCTC	Sigma-Aldrich
HrasQ12/G13/I21 RevI	TCATTTACCCATGACCACTG	Sigma-Aldrich
HrasK117 ForM	GTCCTGGACACAGTCATGC	Sigma-Aldrich
HrasK117 RevN	TGGAGAGGGAAACAGGCTCA	Sigma-Aldrich
HrasW178 ForR	ATTGCCGCTCTCCCTCAAC	Sigma-Aldrich
HrasW178 RevS	TCCTGCCCTAGAGCTATGTGA	Sigma-Aldrich
BrafV584 ForA	TCCTTTACTTACTGCACCTCAG	Sigma-Aldrich
BrafV584 RevAB	CCTGTGAGTAGTGGGAACTGT	Sigma-Aldrich
EgfrF254 ForB1	TGAGCATGTAAAACACCGAAAACT	Sigma-Aldrich
EgfrF254 RevB2	GGTGGCACCAAAGCTGTACT	Sigma-Aldrich
EgfrN758 ForD1	AAGGCAACATGTTACTGGCA	Sigma-Aldrich
EgfrN758 RevD2	GGGTGTAATTTATTAGTGCATCCAG	Sigma-Aldrich
EgfrR778 ForE1	ACCCAGAAAGGGATATGCGTGC	Sigma-Aldrich
EgfrR778 RevE2	GGTACTGGGAGCCAATGTTGT	Sigma-Aldrich
EgfrI928 ForF1	TGGCTAAGGATTAGGTGGTGTG	Sigma-Aldrich
EgfrI928 RevF2	TTCTTCGGGGAAATGAGGGC	Sigma-Aldrich
ApcY156/Y157 ForA1	GCTGAGAGCTACCAGGCAAT	Sigma-Aldrich
ApcY156/Y157 RevA2	GAGTACTCAAAGGTCAAGTTACCA	Sigma-Aldrich
ApcY189 ForB1	GTCAGGGACCCCAGTTAAGC	Sigma-Aldrich
ApcY189 RevB2	AGCAGAGCTGTAGTGTGCAG	Sigma-Aldrich
ApcD326 ForC1	AGCTGTCATCTCTTCATCCAGAA	Sigma-Aldrich
ApcD326 RevC2	CTCTTTACTGCCCCGGGAAT	Sigma-Aldrich
ApcQ1292 ForE1	TGGCCAGACTCAAAAAGGCA	Sigma-Aldrich
ApcQ1292 RevE2	TTCTGGGACACTGCTGGAAC	Sigma-Aldrich
ApcE1450 ForF1	GTGGCATCATAAGCCCCAGT	Sigma-Aldrich
ApcE1450 RevF2	CAGAGCACTTAGGCTGGAGG	Sigma-Aldrich
ApcL1487 ForM	GCCAAGAGAGAGGTGCCAAA	Sigma-Aldrich
ApcL1487 RevM	TTCCTGAACTGGAGGCATGAT	Sigma-Aldrich
ApcN1796 ForG1	TGGACCAAGTCCAACAAGCA	Sigma-Aldrich
ApcN1796 RevG2	CCCCGCACTCTGTCTTCATT	Sigma-Aldrich
ApcS2095 ForH1	GCGAAAAGCAGAGCCCTAGA	Sigma-Aldrich
ApcS2095 RevH2	CTGAGTCGGATGACGCTTGT	Sigma-Aldrich
ApcSA ForI1	TACTTAGCCAGGCTGGAGGT	Sigma-Aldrich
ApcSA RevI2	CCTGACCACTACTGGAGGCT	Sigma-Aldrich

1.5 Biological samples

Description	Source	Identifier

1.6 Deposited data

Name of repository	Identifier	Link
European Nucleotide Archive	PRJEB19083	https://www.ebi.ac.uk/ena/data/view/PRJEB19083 (Not yet public)

European Nucleotide Archive	ERZ537501	(Not yet public)
European Nucleotide Archive	ERZ537503	(Not yet public)
EMBL-EBI Biostudies	S-BSMS4<br>S-BSST141	https://www.ebi.ac.uk/biostudies/

1.7 Software

Software name	Manufacturer	Version
bwa	http://bio-bwa.sourceforge.net/	0.6.1 and 0.7.12
picard	https://broadinstitute.github.io/picard/	1.124
samtools	http://www.htslib.org/	1.1
Strelka	Illumina;<br>https://sites.google.com/site/strelkasomaticvariantcaller/	1.0.14
gatk-tools	CRUK;<br>https://github.com/crukci-bioinformatics/gatk-tools	0.2
bcftools	http://www.htslib.org/	1.1
R	https://www.r-project.org/	3.4.1
boot	https://CRAN.R-project.org/package=boot	1.3-20
ape	https://CRAN.R-project.org/package=ape	3.5
XNomial	https://CRAN.R-project.org/package=XNomial	1.0.4
deconstructSigs	https://CRAN.R-project.org/package=deconstructSigs	1.8.0
reactome.db	https://doi.org/doi:10.18129/B9.bioc.reactome.db	1.62.0
CNVkit	https://github.com/etal/cnvkit	0.7.2

1.8 Other (e.g. drugs, proteins, vectors etc.)

1.9 Please provide the details of the corresponding methods author for the manuscript:

Frances Connor, Frances.Connor@cruk.cam.ac.uk.
Tim Rayner, Tim.Rayner@cruk.cam.ac.uk.

2.0 Please confirm for randomised controlled trials all versions of the clinical protocol are included in the submission. These will be published online as supplementary information.

