# Supplementary material for: Mutational landscape of a chemically-induced mouse model of liver cancer
Source: J Hepatol. 2018 Oct;69(4):840–50. doi: 10.1016/j.jhep.2018.06.009 (PMC6142872; doi:10.1016/j.jhep.2018.06.009)
Supplement: Supplementary data 1 [file mmc1.pdf]

Mutational landscape of a chemically-induced mouse model of liver cancer

Frances Connor, Tim F. Rayner, Sarah J. Aitken, Christine Feig, Margus Lukk,

Javier Santoyo-Lopez, Duncan T. Odom

Table of contents

Supplementary Materials and Methods……………………………………………...2

Fig. S1……………………………………………………………………………….12

Fig. S2……………………………………………………………………………….14

Fig. S3……………………………………………………………………………….16

Fig. S4……………………………………………………………………………….18

Table S1. Legend…………………………………………………………………..19

Table S2…………………………………………………………………………… 20

Table S3…………………………………………………………………………… 22

Supplementary references………………………………………………………..24

Supplementary Materials and Methods

Mouse models of hepatocarcinogenesis

All animal experimentation was carried out in accordance with the Animals (Scientific Procedures) Act 1986 (United Kingdom) and with the approval of the Cancer Research UK Cambridge Institute Animal Welfare and Ethical Review Body. Animal experiments conformed to the Animal Research: Reporting of *In Vivo* Experiments (ARRIVE) guidelines developed by the National Centre for the Replacement, Refinement and Reduction of Animals in Research (NC3Rs). C3H/HeOuJ strain mice were obtained from Charles River Laboratories and maintained using standard husbandry. Mice were housed in Tecniplast GM500 Mouse IVC Green Line cages in a room with 12 hour light / 12 hour dark cycles. When possible, animals were group housed. Cages contained aspen bedding and the following cage enrichments: nesting material, aspen chew stick and cardboard tunnel. Mice had *ad libitum* access to water and food (LabDiet 5058).

Liver tumours were chemically induced in male mice aged 14-16 days by a single intraperitoneal injection of diethylnitrosamine (DEN; Sigma-Aldrich N0258; 20mg/kg body weight) diluted in 0.85% saline. To assess the immediate response to DEN, some mice were humanely killed at 4, 8, 12, and 24 hours after DEN injection. Liver tumour samples were collected from DEN-treated mice aged for 24 to 40 weeks after injection. A separate cohort of untreated male mice was aged for 37 to 76 weeks to develop spontaneous liver neoplasms.

Tissue collection, processing, and staining

Ear and tail samples from untreated mice were flash frozen for use as a normal genome reference. Liver tumours were macroscopically identified and isolated. Small nodules were flash frozen in liquid nitrogen for subsequent DNA extraction. Nodules of sufficient size (>2mm diameter) were bisected; one half was flash frozen in liquid nitrogen for DNA extraction and the other half was fixed in neutral buffered formalin for 24 hours, transferred to 70% ethanol, machine processed (Leica ASP300 Tissue Processor) and paraffin embedded. Normal liver tissue from untreated, adult C3H male mice was fixed in neutral buffered formalin and processed as described for the nodule tissue. All formalin-fixed paraffin-embedded (FFPE) sections were 3 µm in thickness.

FFPE tissue sections were stained with haematoxylin and eosin (H&E) for morphological assessment and using the Gomori's method for reticular fibres to assess liver architecture. Histochemical staining was performed using the automated Leica ST5020; mounting was performed on the Leica CV5030. Immunohistochemistry was performed on FFPE tissue sections using the Bond Polymer Refine Kit (DS9800, Leica Microsystems) with DAB enhancer (Leica Biosystems, AR9432) on the automated Bond platform. Immunohistochemistry was performed using antibodies against β-catenin (BD Biosciences, 610154, 1:100 dilution); phospho-histone H2AX (Merck Millipore, MABE205, 1:5000); O^6-ethyl-2-deoxyguanosine (ER6, Squarix Biotechnology, SQM001.1, 1:200); Ki67 (Bethyl Laboratories, IHC-00375, 1:1000) and CD45 (R&D Systems, MAB1217, 1:750). Heat-induced epitope retrieval was performed for 10 minutes (CD45 only) or 20 minutes at 100°C on the Bond platform with sodium citrate. For the anti-O^6-ethyl-2-deoxyguanosine staining the peroxidase block was done post-primary antibody

incubation and the post-primary component of the polymer kit was substituted with a rabbit anti-rat secondary antibody (Bethyl Laboratories, A110-322A, 1:250). All tissue sections were digitised at x20 magnification using an automated scanning system, Aperio XT (Leica Biosystems), and visualised using the ImageScope software (Leica Biosystems); H&E-stained tissue section images are available at BioStudies archive at EMBL-EBI under accession S-BSMS4 and S-BSST141.

Quantification of nuclear staining for O^6-ethyl-2-deoxyguanosine and phospho-histone H2AX was done using digitised images with ImageScope software. On average 505,000 cells from one cross-section per sample were evaluated (range: 290,000 – 690,000). Percent of positive-staining nuclei were plotted using the ggplot2 package in RStudio and statistical significance was calculated using the Welch two sample t-test.

Histological classification and selection of liver tumours

Tissue sections were blinded and assessed twice by a histopathologist; discordant results were reviewed by an independent hepatobiliary histopathologist. Tumours were classified according to the International Harmonization of Nomenclature and Diagnostic Criteria for Lesions in Rats and Mice (INHAND) guidelines [1]. Dysplastic nodules (DNs) have an expansile growth pattern causing compression of adjacent hepatic parenchyma, loss of normal lobular architecture (irregular reticulin fibre staining), nuclear atypia, and may show increased proliferation (increased Ki67 staining). Hepatocellular carcinomas (HCCs) are characterised by thickened trabeculae (loss of reticulin fibre staining), pseudoglandular structures, more marked

cellular atypia, increased nuclear to cytoplasmic ratios, higher proliferative index (markedly increased Ki67 staining) and an infiltrative growth pattern.

Tumours with sufficient tissue for histological classification were selected for sequencing experiments if they met the following criteria: (i) diagnosis of either DN or HCC, (ii) homogenous tumour morphology, (iii) tumour cell percentage >80%, and (iv) adequate tissue for DNA extraction. Neoplasms with extensive necrosis, mixed tumour types, a nodule-in-nodule appearance (indicative of an HCC which had arisen within a DN), or contamination by normal liver tissue were excluded.

DNA isolation and whole exome sequencing

Genomic DNA from liver tumours was isolated using the AllPrep DNA/RNA Mini Kit (Qiagen, 80204). Genomic DNA from ear/tail samples was extracted with the DNeasy Blood & Tissue Kit (Qiagen, 69506). Exome capture libraries were prepared following the instructions of the SureSelectXT Mouse All Exon Target Enrichment System (Agilent Technologies, 5190-4641). Briefly, 3µg DNA were sheared to 150-300bp fragments using the Covaris S220 system. Libraries were generated with the SureSelectXT reagent kit (G9611A), hybridised and enriched according to the manufacturer's instructions. During this process all clean-up steps were performed with Agencourt AMPure XP beads (Beckman Coulter, A63880), all DNA concentration measurements were done with the Qubit fluorometric method (ThermoFisher) and library quality controls were run on the Agilent Bioanalyzer 2100 (DNA1000, DNA high-sensitivity). Finally, the exome libraries were quantified by real-time PCR using the Kapa library quantification kit (KapaBiosystems) on the

QuantStudio 6 Flex (Applied Biosystems) before pooling and sequencing 125bp paired-end reads on an Illumina HiSeq2500.

Sequencing read alignment

Sequencing reads were aligned to the C3H_HeJ_v1 mouse genome assembly (Ensembl release 90 [2]) with bwa (versions 0.6.1 or 0.7.12 [3]). Aligned reads were annotated to read groups using the picard tool AddOrReplaceReadGroups and minor annotation inconsistencies corrected using the picard CleanSam and FixMateInformation tools (picard version 1.124; [4]). The bam files for each sample were merged together and duplicate reads were then identified using the picard MarkDuplicates tool. Sequencing coverage was assessed using samtools (version 1.1 [5]). 95% of genome regions predicted to represent coding sequences were found to be covered to a read depth of 20 or greater. Raw sequencing reads are available from European Nucleotide Archive at EMBL-EBI under accession PRJEB19083. Aligned reads for human samples from the LICA-FR and LIAD-FR cohorts were downloaded from the European Genome-phenome Archive at EMBL-EBI ([6]; accessions EGAD00001000131, EGAD00001001096 and EGAD00001000737; access permission reference DACO-1021377).

Variant calling

A pooled normal sample set was generated by combining all control sample reads and then subsampling these reads to match the mean coverage achieved for the control and tumour samples. Single nucleotide and indel variants were called using Strelka (version 1.0.14 [7]) using the recommended configuration for bwa-aligned reads and setting the isSkipDepthFilters flag for improved calling on exome-seq

data. Variant calls were combined into a merged set using bcftools (version 1.1 [5]). Predicted coding sequence changes due to SNVs were annotated by comparing the reference polypeptide sequences to variant alleles.

SNVs were subjected to multiple filtering steps. Firstly, low-confidence SNV calls were identified and removed by applying the recommended filters for Strelka output from the gatk-tools package (version 0.2 [8]; [9]). In particular, variants were filtered based on low mapping and base quality scores, proximity to alignment ends, and low absolute read counts. VCF files are available from European Nucleotide Archive at EMBL-EBI under accessions ERZ537501 and ERZ537503. Secondly, SNVs with an allele frequency of less than 2.5% were omitted to eliminate possible cross-contamination due to observed low levels of sequence read index misassignment during Illumina sequencing [10].

SNV call rates were estimated by fitting the number of variants detected at a range of sequencing read depths and extrapolating to determine the expected call rate at saturated sequencing coverage. Variant allele frequencies were calculated from read counts for reference and variant alleles, excluding those reads having a MAPQ score less than 5. Confidence intervals for allele frequency estimation were estimated by applying a normal distribution approximation to bootstrap-resampled frequency estimates (R boot package version 1.3 [11]).

Autosomal copy number variations (CNVs) were called with CNVkit (version 0.7.2 [12]) using default parameters. CNV regions were filtered to remove low-confidence regions where the null hypothesis (i.e., unchanged copy number) fell within the 95%

confidence interval. Further filters removed CNVs where the absolute log fold change in copy number was smaller than 0.25. CNV regions located within 10kb of each other were merged, and the resulting regions finally filtered to remove regions smaller than 10Mb.

SNV validation

Non-synonymous SNVs in the cancer driver genes of interest, *Hras*, *Braf*, *Egfr* and *Apc*, were validated using conventional Sanger sequencing. The majority (87%) were confirmed using this method. The remaining SNVs could not be validated by Sanger sequencing for technical reasons because either there was no sample remaining or the variant allele frequency was too low to allow confident detection by this method. These SNVs were checked by visual inspection of the aligned reads and were called validated if the total variant reads were greater than ten. The method used for each SNV validation is noted in Table S3.

Independence of tumour evolution

For phylogenetic and mutational signature analyses (see below) the SNV list was filtered to remove those genome regions which were not covered by at least 20 non-duplicated reads (i.e. coverage of 20x or above) across all samples. This yielded a data set in which samples may be reasonably assumed to bear homozygous reference alleles at the vast majority of loci for which Strelka does not call a variant. Protein coding, exonic, and genic regions represented 42.6%, 53.7% and 92.0% of these 20x covered genomic regions, respectively.

A phylogenetic tree describing the development of the tumours within our cohorts was constructed in R using the ape package (version 3.5 [13]). Pairwise distances between samples were calculated as the number of genomic loci at which the sample genotypes differ. Trees were constructed using a neighbour-joining algorithm [14].

Mutational signature analysis

Analysis of mutational signatures was constrained to just those regions covered to at least 20x in all samples. The distributions of 5' and 3' nucleotides flanking the called SNVs were calculated directly from the reference genome. Direct comparison between human and mouse signatures was facilitated by normalising C3H/HeJ nucleotide context distributions using the ratios of known trinucleotide frequencies in C3H/HeJ and human genomes, as calculated for the 20x covered regions for each genome.

The proportions of COSMIC mutational signatures [15] represented in the mutational profile from each sample was calculated using the R package deconstructSigs (version 1.8.0 [16]).

Identification of significantly mutated cancer-related genes

Variants were annotated as to their likely effect on coding sequence by comparing their predicted polypeptide sequences to those from the Ensembl release 90 C3H_HeJ_v1 reference. SNV calls which were shared between tumours taken from the same mouse (i.e. SNVs which could be simply ascribed to germline variation) were filtered out prior to analysis of mutated genes.

Cancer-related genes bearing above expected levels of non-synonymous mutations (both across the entire gene and recurring at specific loci) were identified using the following procedure. The gene list to be analysed was constrained to the listing of oncogenes and tumour suppressor genes described by Vogelstein *et al*., 2013 [17]. The total mutation load (nonsense, missense, frame shift, splice site) within coding and splice-site regions was used to calculate the probability that mutations had occurred purely by stochastic mutational processes. Within each gene, the count of mutations at each variant locus was fitted to a Poisson distribution assuming a background mutation rate calculated across all sequenced regions. The individual variant loci were combined at the gene level using a multinomial model using the R XNomial package (version 1.0.4; [18]). This model yielded log likelihood ratios from the observed and expected distributions, from which a gene-wise p-value was readily calculated. P-values were corrected for multiple testing across all genes using the Bonferroni method. The analysis was repeated imposing a gene filter derived from the Cosmic Cancer Gene Census [19]. This identified the same cancer-associated genes that were significantly mutated in the mouse neoplasms.

Clustering of human and mouse samples by Reactome pathway analysis

Cancer-associated genes [19] containing non-synonymous SNVs were used to annotate each sample with a list of affected Reactome pathways (R reactome.db package, version 1.62.0; [20]). Pairwise distances between samples were calculated from the numbers of differentially mutated pathways in each case. The distances between sample clusters were visualised by constructing a tree using a neighbour-joining algorithm [13].

Generation and analysis of DEN-initiated tumour samples from C57BL/6J mice

The supplementary data for the mouse strain comparison are from DEN-induced liver tumours which were generated as part of the control cohort for a separate study. The mice were congenic on the C57BL/6J background and carried one conditional *Ctcf* allele [21]; these mice have been reported to be phenotypically normal with CTCF expression equivalent to wild-type mice. Livers tumours were chemically induced using DEN, collected and classified using the same protocols as described for C3H mice. Genomic DNA (gDNA) was isolated using the AllPrep DNA/RNA 96 Kit (Qiagen 80311) according to the manufacturer's instructions. 1µg gDNA was used to generate whole genome sequencing libraries using the TruSeq PCR-free Library Prep Kit (Illumina) according to the manufacturer's instructions. Libraries were diluted to 0.75nM and pooled libraries were sequenced on an Illumina HiSeq X Ten according to manufacturer's instructions to produce paired-end 150bp reads. Whole genome sequencing reads were aligned to the GRCm38 genome assembly (Ensembl release 90 [2]) with bwa (version 0.7.12 [3]). Single nucleotide variant calling was limited to regions in direct synteny with the 20x covered regions which were identified for analysis in our C3H/HeOuJ cohort. Mutational signature and driver gene analyses were carried out using the same procedures as described for the C3H/HeOuJ sequencing data.

Fig. S1. Tumour infiltrating lymphocytes are not a prominent feature of DEN-initiated and spontaneous mouse liver tumours.

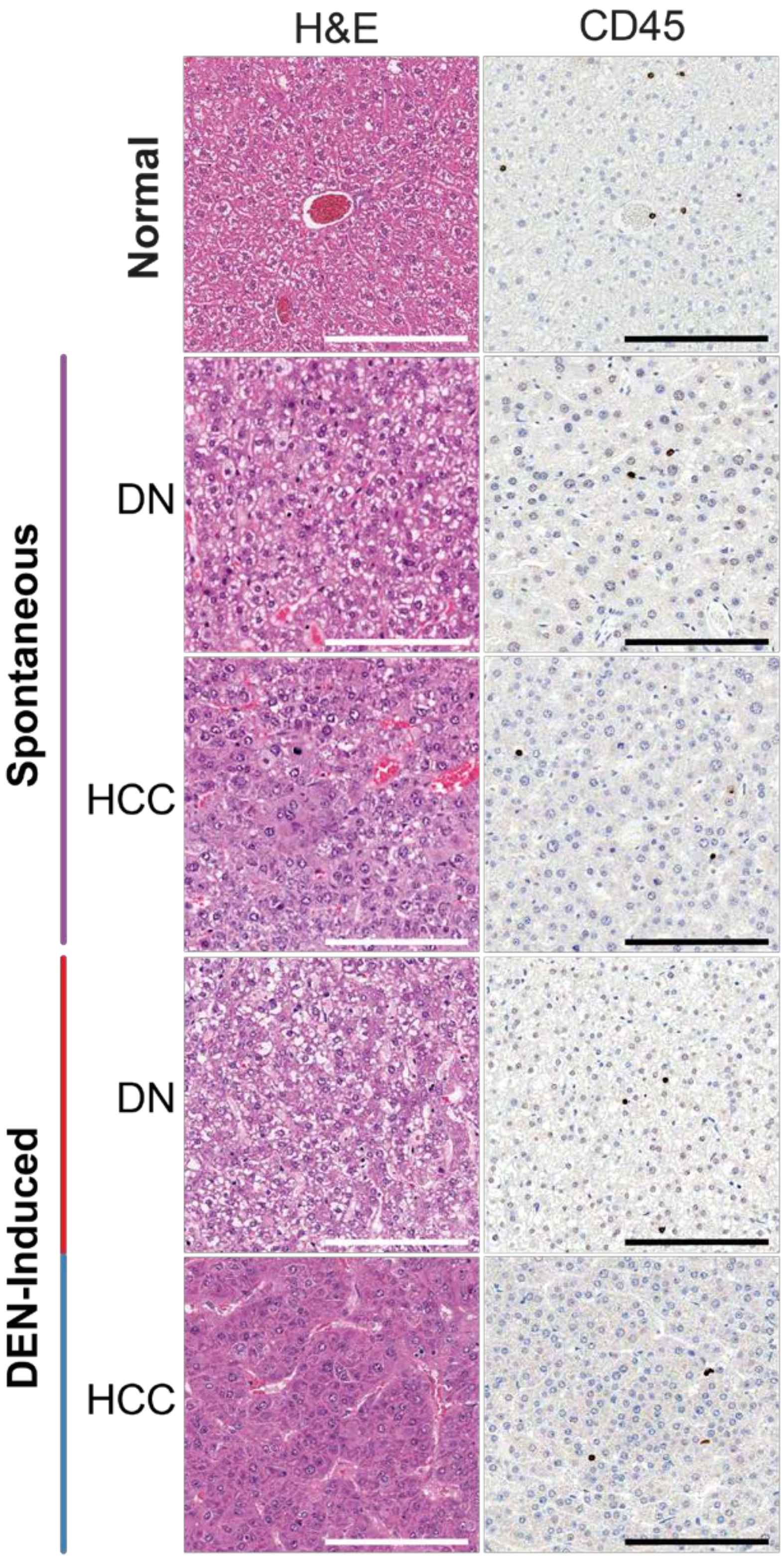

Representative photomicrographs of serial tissue sections from C3H mice: normal liver tissue from untreated adult (27 weeks) mice; spontaneous tumours arising in untreated mice; and DEN-initiated tumours (DN: dysplastic nodule; HCC: hepatocellular carcinoma). Histochemical staining for CD45 is shown on the right and H&E staining of the corresponding serial sections is shown on the left. All scale bars = 200μm. Original magnification x200.

Fig. S2. Exposure to DEN leaves a distinct and reproducible mutational imprint in liver tumours arising in both C3H and B6 mice.

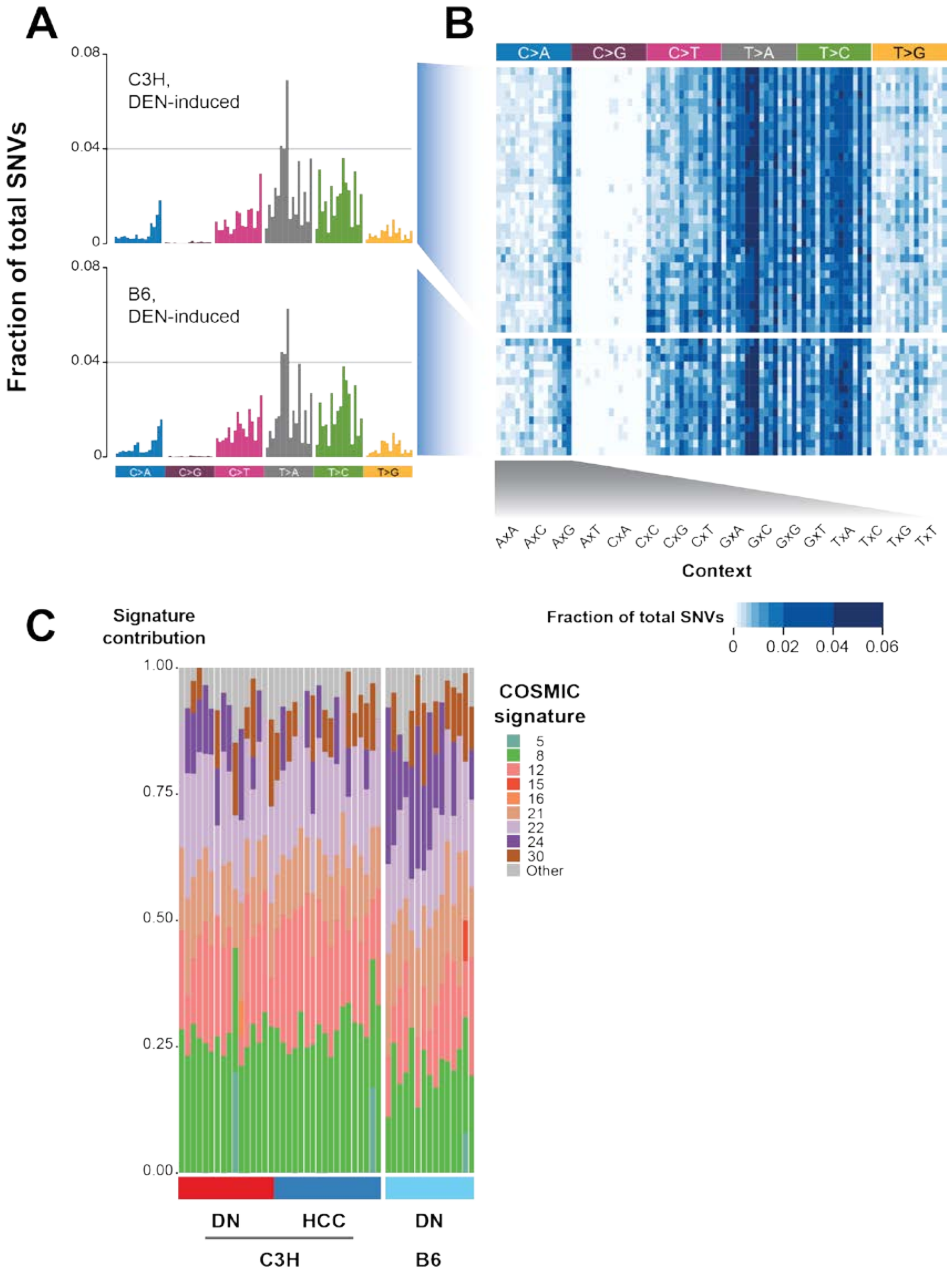

(A) Frequencies of single nucleotide substitutions, displayed using the 96 substitution classification, in DEN-induced liver tumour cohorts from C3H/HeOuJ and C57BL/6J mice (n= 34 tumours from 25 C3H mice; n= 15 tumours from 6 B6 mice).

(B) Heat map of the occurrence of mutational profiles of individual DEN-induced liver tumours in C3H and B6 mice (rows) classified by substitution and trinucleotide context (columns).

(C) Mutational portraits of individual DEN-induced tumours in C3H and B6 mice reconstructed using COSMIC mutational signatures. Each column shows the composition of signatures in an individual sample (DN, dysplastic nodule; HCC, hepatocellular carcinoma).

Fig. S3. Mouse strain influences the high prevalence of activating mutations in either *Hras* or *Braf* in DEN-initiated liver tumours.

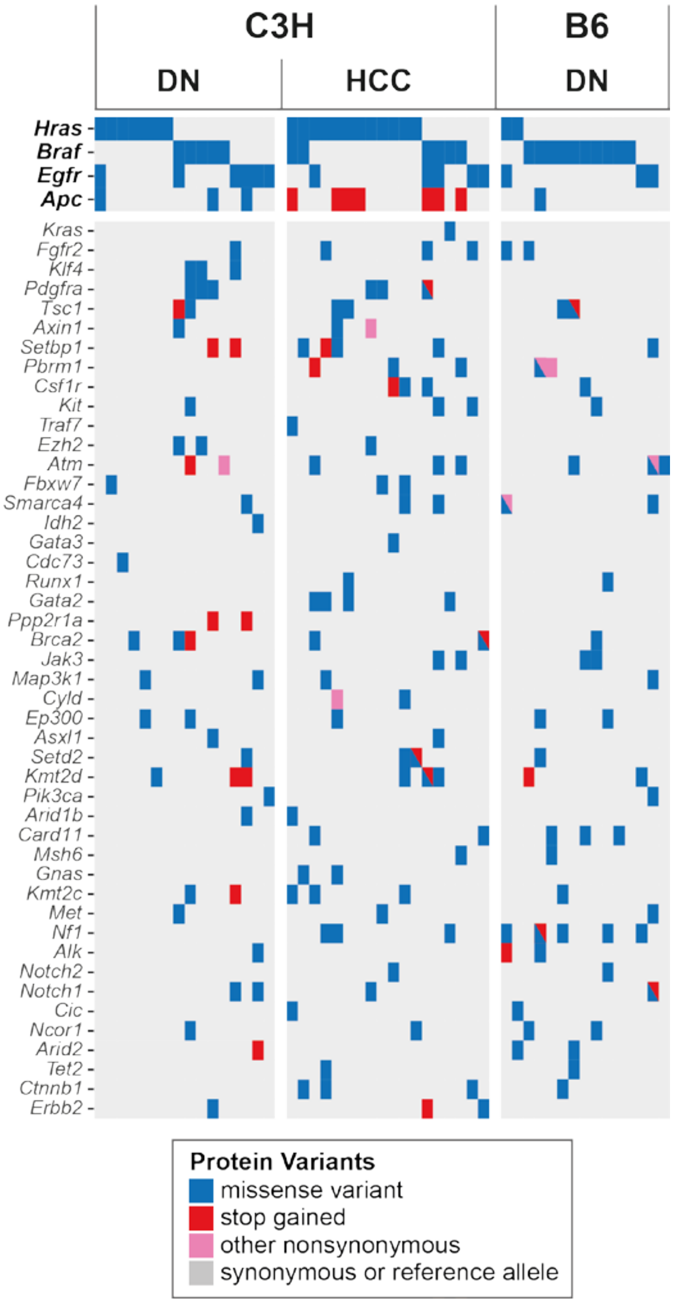

Predicted consequential mutations in oncogenes and tumour suppressor genes for individual DEN-induced tumours in C3H/HeOuJ and C57BL/6J mice. Each column is a tumour sample (DN, dysplastic nodule; HCC, hepatocellular carcinoma) and each

row is a cancer gene showing the occurrence of non-synonymous substitutions found in individual samples. Only genes mutated in at least two samples are shown.

Fig. S4. Pathway analysis of cancer genomes highlights distinct characteristics of liver tumours in humans compared to those arising in spontaneous and DEN-induced mouse models.

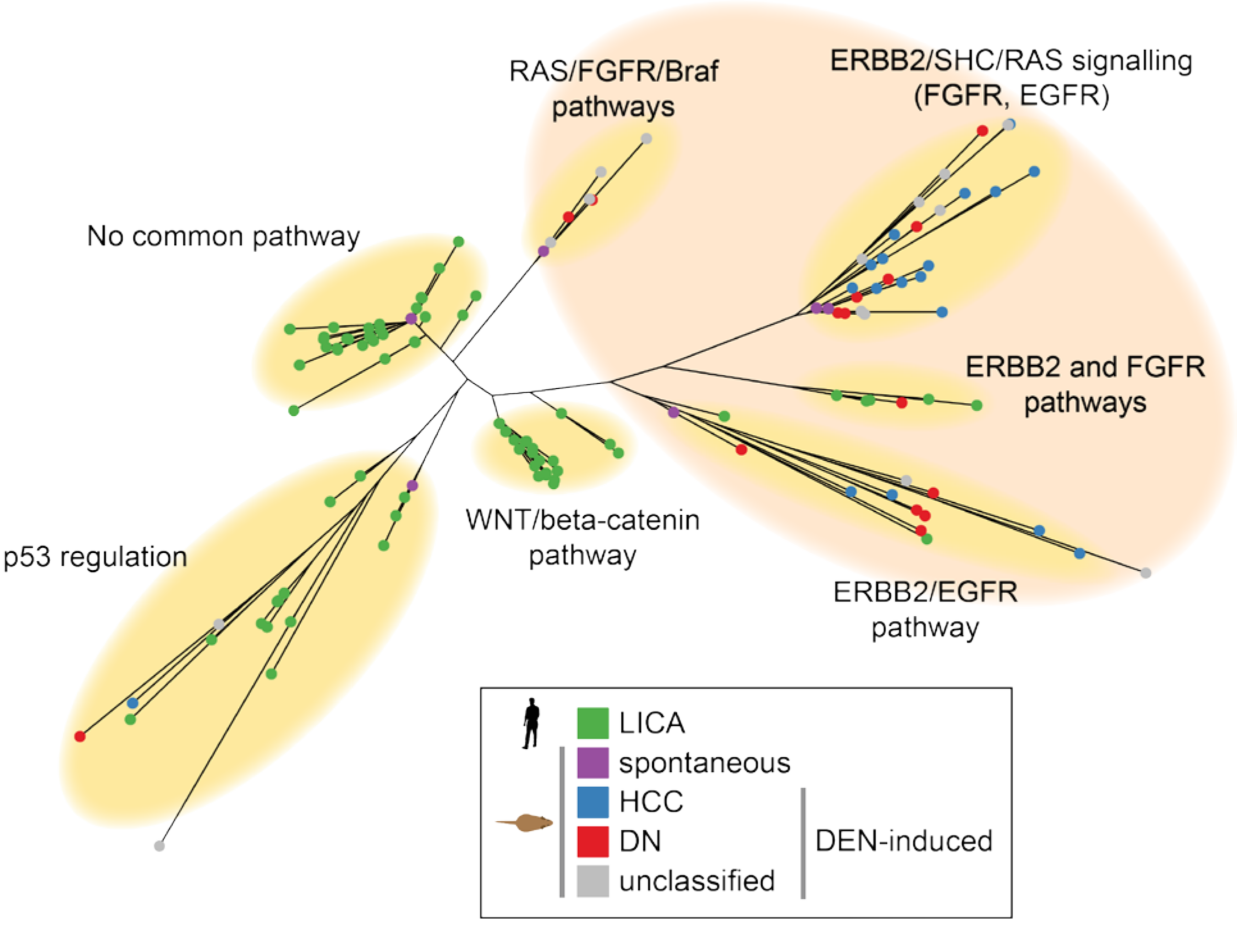

Each dot represents a liver tumour sample from human (LICA) or from mouse (C3H/HeOuJ; spontaneous or DEN-induced). Samples were annotated based on the presence of Reactome pathways with a component cancer-associated gene(s) carrying a non-synonymous SNV. Samples were then clustered according to pairwise distances calculated from the numbers of differentially mutated pathways between samples.

Table S1 Legend.

Somatic non-synonymous SNVs detected in cancer driver genes in DEN-induced and spontaneous mouse liver tumours.

Individual liver tumour samples are arranged in columns using the mouse and tumour identification codes (DN: DEN-induced dysplastic nodule; HCC: DEN-induced hepatocellular carcinoma; spontaneous: tumour from an untreated mouse). Details of the SNVs in oncogenes and tumour suppressor genes are shown in the rows. SNVs are coded as "0/1" to indicate a detected heterozygous variant genotype, and "." to indicate no variant call. Predictions of the likely consequences of mutations were incorporated as follows: genomic locations of the SNVs were lifted over from the C3H_HeJ_v1 genome assembly to the GRCm38 mouse assembly; the resulting loci were used to query PROVEAN, SIFT and COSMIC database tools.

Table S2. Occurrence of non-synonymous SNVs in *Hras*, *Braf*, *Egfr* and *Apc* in dysplastic nodules (DN) and hepatocellular carcinomas (HCC) from livers of DEN-treated and untreated C3H mice.

Gene	Mutation	Number of neoplasms with the specified mutation			
		**DEN-initiated DN** (n=16)	**DEN-initiated HCC** (n=18)	**Spontaneous DN** (n=22)	**Spontaneous HCC** (n=3)
Hras	G12L	0	0	1	0
Hras	G13R	0	0	0	1
Hras	G13V	0	0	3	0
Hras	I21N	0	1	0	0
Hras	Q61K	1	2	0	0
Hras	Q61L	0	4[a,c,d]	1	0
Hras	Q61R	6[g]	5	0	0
Hras	K117N	0	0	6	0
Hras	W178R	0	1[a]	0	0
Braf	D578G	1[b]	0	0	0
Braf	V584E	5[b]	6[c,e,f]	1	0
Egfr	F254I	4	5[d,e]	0	0
Egfr	N758K	1[f]	0	0	0
Egfr	R778H	0	0	1	0
Egfr	I928N	1[g]	0	0	0
Apc	Y156*	0	1	0	0
Apc	Y157*	0	1	0	0
Apc	Y189*	0	1	0	0
Apc	spl?[h]	0	1	0	0
Apc	D326G	1	0	0	0
Apc	L850*	0	1	0	0
Apc	Q1292*	0	1	0	0
Apc	E1450*	0	1	0	0
Apc	L1487*	0	1	0	0
Apc	N1796K	1	0	0	0
Apc	S2095P	1	0	0	0

[a] *Hras* W178R co-occurred with *Hras* Q61L in one DEN-initiated HCC sample.

[b] *Braf* D578G co-occurred with *Braf* V584E in one DEN-initiated DN sample.

[c] *Braf* V584E co-occurred with *Hras* Q61L in one DEN-initiated HCC sample.

[d] *Egfr* F254I co-occurred with *Hras* Q61L in one DEN-initiated HCC sample.

[e] *Egfr* F254I co-occurred with *Braf* V584E in two DEN-initiated HCC samples.

[f] *Egfr* N758K co-occurred with *Braf* V584E in one DEN-initiated DN sample.

[g] *Egfr* I928N co-occurred with *Hras* Q61R in one DEN-initiated DN sample.

[h] spl?, splice acceptor variant that might affect splicing.

Table S3. Non-synonymous SNVs in *Hras*, *Braf*, *Egfr* and *Apc* in C3H mouse liver neoplasms.

Gene	Chr	Genome Coordinate	Allele REF	Allele ALT	Peptide Variant	Sample	VAF	Validated	Comment
Hras	chr7	145859242	T	C	Q61R	90796_N1	0.472	Sanger sequencing	
		145859242	T	C	Q61R	89986_N3	0.452	Sanger sequencing	
		145859242	T	A	Q61L	87187_C6	0.447	Sanger sequencing	
		145859242	T	C	Q61R	89073_C3	0.440	Sanger sequencing	
		145859243	G	T	Q61K	90997_C7	0.420	Sanger sequencing	
		145859242	T	C	Q61R	86772_C6	0.419	Sanger sequencing	
		145858922	C	A	K117N	87922_N2	0.406	Sanger sequencing	
		145859242	T	C	Q61R	93091_N1	0.406	Inspection of reads	no sample remaining
		145859596	C	G	G13R	87922_N3	0.396	Sanger sequencing	
		145859242	T	C	Q61R	91099_N1	0.395	Sanger sequencing	
		145859242	T	A	Q61L	90796_N4	0.394	Sanger sequencing	
		145859243	G	T	Q61K	92188_N3	0.388	Sanger sequencing	
		145858922	C	A	K117N	96029_N2	0.376	Sanger sequencing	
		145858647	A	T	W178R	87187_C6	0.375	Sanger sequencing	
		145859242	T	C	Q61R	87104_C1	0.372	Sanger sequencing	
		145859242	T	A	Q61L	96988_N1	0.372	Sanger sequencing	
		145859242	T	C	Q61R	87187_C5	0.364	Sanger sequencing	
		145858922	C	A	K117N	97732_N1	0.363	Sanger sequencing	
		145859242	T	C	Q61R	92186_N2	0.348	Sanger sequencing	
		145859598	CC	AA	G12L	87920_N3	0.348	Sanger sequencing	
		145858922	C	A	K117N	87918_N1	0.345	Sanger sequencing	
		145859242	T	C	Q61R	93258_N4	0.345	Sanger sequencing	
		145858922	C	A	K117N	87920_N2	0.331	Sanger sequencing	
		145859571	A	T	I21N	87574_C3	0.331	Sanger sequencing	
		145859242	T	C	Q61R	93091_N3	0.328	Sanger sequencing	
		145859243	G	T	Q61K	87574_C5	0.309	Sanger sequencing	
		145859595	C	A	G13V	92759_N1	0.304	Sanger sequencing	
		145858922	C	A	K117N	87920_N1	0.268	Sanger sequencing	
		145859595	C	A	G13V	96504_N1	0.224	Inspection of reads	no sample remaining
		145859595	C	A	G13V	92757_N1	0.200	Inspection of reads	no sample remaining
		145859242	T	A	Q61L	90997_C9	0.147	Inspection of reads	low VAF
		145859242	T	A	Q61L	87575_C1	0.085	Inspection of reads	low VAF
Braf	chr6	37548568	A	T	V584E	91596_C4	0.414	Sanger sequencing	
		37548568	A	T	V584E	87574_C3	0.409	Sanger sequencing	
		37548568	A	T	V584E	93129_N7	0.383	Sanger sequencing	
		37548568	A	T	V584E	90793_N2	0.360	Sanger sequencing	
		37548568	A	T	V584E	93091_N8	0.357	Sanger sequencing	
		37548568	A	T	V584E	86772_C1	0.340	Sanger sequencing	
		37548568	A	T	V584E	91100_N3	0.337	Sanger sequencing	
		37548568	A	T	V584E	89072_C4	0.306	Sanger sequencing	
		37548568	A	T	V584E	90997_C9	0.272	Sanger sequencing	
		37548568	A	T	V584E	91415_N3	0.247	Sanger sequencing	
		37548568	A	T	V584E	92189_N4	0.159	Sanger sequencing	
		37548568	A	T	V584E	87922_N1	0.085	Inspection of reads	low VAF
		37548586	T	C	D578G	90793_N2	0.053	Inspection of reads	low VAF
Egfr	chr11	14185624	T	A	F254I	91930_N1	0.441	Sanger sequencing	
		14185624	T	A	F254I	91419_C8	0.408	Sanger sequencing	
		14185624	T	A	F254I	87187_C7	0.387	Sanger sequencing	
		14210588	C	A	N758K	93091_N8	0.349	Sanger sequencing	
		14185624	T	A	F254I	90796_N2	0.345	Sanger sequencing	
		14224314	T	A	I928N	93258_N4	0.333	Sanger sequencing	
		14185624	T	A	F254I	90022_N2	0.322	Sanger sequencing	
		14185624	T	A	F254I	93100_N6	0.295	Sanger sequencing	
		14185624	T	A	F254I	87575_C1	0.267	Sanger sequencing	
		14214433	G	A	R778H	85037_N6	0.195	Sanger sequencing	
		14185624	T	A	F254I	92189_N4	0.145	Sanger sequencing	
		14185624	T	A	F254I	89072_C4	0.070	Inspection of reads	low VAF
Apc	chr18	32288741	T	A	Y156*	90796_N4	0.901	Sanger sequencing	
		32333453	C	T	Q1292*	87104_C1	0.812	Sanger sequencing	
		32294140	T	G	Y189*	87187_C5	0.660	Sanger sequencing	
		32334039	T	A	L1487*	91596_C4	0.788	Sanger sequencing	
		32334967	T	A	N1796K	93100_N6	0.473	Sanger sequencing	
		32332128	T	A	L850*	89072_C4	0.385	Inspection of reads	technical failure
		32288744	T	A	Y157*	90997_C9	0.384	Sanger sequencing	
		32335862	T	C	S2095P	93258_N4	0.352	Sanger sequencing	
		32307542	C	A	r.spl?	87187_C6	0.306	Sanger sequencing	
		32316102	A	G	D326G	91415_N3	0.304	Sanger sequencing	
		32333927	G	T	E1450*	92189_N4	0.286	Sanger sequencing	

Legend: Sanger sequencing (green); Inspection of reads (yellow)

The tumour samples carrying a non-synonymous SNV in *Hras*, *Braf*, *Egfr* and/or *Apc* are listed. Details of the individual SNVs, including genome coordinate, nucleotide and peptide variant, variant allele frequency (VAF) and validation method, are shown for each liver tumour sample. Variants were validated by Sanger sequencing whenever possible, or by visual inspection of aligned reads.

Supplementary References

Author names in bold designate shared co-first authorship.

[1] Thoolen B, Maronpot RR, Harada T, Nyska A, Rousseaux C, Nolte T, et al. Proliferative and nonproliferative lesions of the rat and mouse hepatobiliary system. Toxicol Pathol 2010;38:5S-81S.

[2] Yates A, Akanni W, Amode MR, Barrell D, Billis K, Carvalho-Silva D, et al. Ensembl 2016. Nucleic Acids Res 2016;44:D710-716.

[3] Li H, Durbin R. Fast and accurate long-read alignment with Burrows-Wheeler transform. Bioinformatics 2010;26:589-595.

[4] Picard Tools. [cited 2018]. Available from: http://broadinstitute.github.io/picard

[5] **Li H**, **Handsaker B**, Wysoker A, Fennell T, Ruan J, Homer N, et al. The Sequence Alignment/Map format and SAMtools. Bioinformatics 2009;25:2078-2079.

[6] European Genome-Phenome Archive. [cited 2018]. Available from: https://ega-archive.org/

[7] Saunders CT, Wong WS, Swamy S, Becq J, Murray LJ, Cheetham RK. Strelka: accurate somatic small-variant calling from sequenced tumor-normal sample pairs. Bioinformatics 2012;28:1811-1817.

[8] **Alioto TS**, **Buchhalter I**, Derdak S, Hutter B, Eldridge MD, Hovig E, et al. A comprehensive assessment of somatic mutation detection in cancer using whole-genome sequencing. Nat Commun 2015;6:10001.

[9] gatk-tools [cited 2018]. Available from: https://github.com/crukci-bioinformatics/gatk-tools

[10] Vodak D, Lorenz S, Nakken S, Aasheim LB, Holte H, Bai B, et al. Sample-index misassignment impacts tumour exome sequencing. Sci Rep 2018;8:5307.

[11] Davison AC, Hinkley DV. Bootstrap Methods and their Application: Cambridge University Press; 1997.

[12] Talevich E, Shain AH, Botton T, Bastian BC. CNVkit: Genome-wide copy number detection and visualization from targeted DNA sequencing. PLoS Comput Biol 2016;12:e1004873.

[13] Paradis E, Claude J, Strimmer K. APE: Analyses of Phylogenetics and Evolution in R language. Bioinformatics 2004;20:289-290.

[14] Saitou N, Nei M. The neighbor-joining method: a new method for reconstructing phylogenetic trees. Mol Biol Evol 1987;4:406-425.

[15] Forbes SA, Beare D, Boutselakis H, Bamford S, Bindal N, Tate J, et al. COSMIC: somatic cancer genetics at high-resolution. Nucleic Acids Res 2017;45:D777-D783.

[16] Rosenthal R, McGranahan N, Herrero J, Taylor BS, Swanton C. DeconstructSigs: delineating mutational processes in single tumors distinguishes DNA repair deficiencies and patterns of carcinoma evolution. Genome Biol 2016;17:31.

[17] Vogelstein B, Papadopoulos N, Velculescu VE, Zhou S, Diaz LA, Jr., Kinzler KW. Cancer genome landscapes. Science 2013;339:1546-1558.

[18] XNomial. [cited 2018]. Available from: https://CRAN.R-project.org/package=XNomial

[19] Cosmic Cancer Gene Census. [cited 2018]. Available from: http://cancer.sanger.ac.uk/census/

[20] reactome.db. [cited 2018]. Available from: https://doi.org/doi:10.18129/B9.bioc.reactome.db

[21] **Heath H**, **Ribeiro de Almeida C**, Sleutels F, Dingjan G, van de Nobelen S, Jonkers I, et al. CTCF regulates cell cycle progression of alphabeta T cells in the thymus. EMBO J 2008;27:2839-2850.
